# In Situ Defect Engineering Route to Optimize the Cationic Redox Activity of Layered Double Hydroxide Nanosheet via Strong Electronic Coupling with Holey Substrate

**DOI:** 10.1002/advs.202103368

**Published:** 2021-10-28

**Authors:** Xiaoyan Jin, Taehun Lee, Wilson Tamakloe, Sharad B. Patil, Aloysius Soon, Yong‐Mook Kang, Seong‐Ju Hwang

**Affiliations:** ^1^ Department of Materials Science and Engineering College of Engineering Yonsei University Seoul 03722 Republic of Korea; ^2^ Center for Artificial Synesthesia Materials Discovery Department of Materials Science and Engineering Yonsei University Seoul 03722 Republic of Korea; ^3^ Department of Materials Science and Engineering Korea University Seoul 02841 Republic of Korea; ^4^ Department of Chemistry and Nanoscience College of Natural Sciences Ewha Womans University Seoul 03760 Republic of Korea; ^5^ KU‐KIST Graduate School of Converging Science and Technology Korea University Seoul 02841 Republic of Korea

**Keywords:** cationic redox activity, defect engineering, electrocatalyst, in situ analysis, layered double hydroxides

## Abstract

A defect engineering of inorganic solids garners great deal of research activities because of its high efficacy to optimize diverse energy‐related functionalities of nanostructured materials. In this study, a novel in situ defect engineering route to maximize electrocatalytic redox activity of inorganic nanosheet is developed by using holey nanostructured substrate with strong interfacial electronic coupling. Density functional theory calculations and in situ spectroscopic analyses confirm that efficient interfacial charge transfer takes place between holey TiN and Ni−Fe‐layered double hydroxide (LDH), leading to the feedback formation of nitrogen vacancies and a maximization of cation redox activity. The holey TiN−LDH nanohybrid is found to exhibit a superior functionality as an oxygen electrocatalyst and electrode for Li−O_2_ batteries compared to its non‐holey homologues. The great impact of hybridization‐driven vacancy introduction on the electrochemical performance originates from an efficient electrochemical activation of both Fe and Ni ions during electrocatalytic process, a reinforcement of interfacial electronic coupling, an increase in electrochemical active sites, and an improvement in electrocatalysis/charge‐transfer kinetics.

## Graphical abstract

Here the development of a novel synthetic route to defect‐optimized high‐performance electrocatalyst via the hybridization‐driven in situ creation of crystal defects, is reported. The holey TiN−LDH nanohybrid with strong interfacial electronic coupling shows outstanding oxygen electrocatalytic activity for Li−O_2_ batteries, which is attributable to the feedback formation of nitrogen vacancies and the maximization of cation redox activity upon hybridization.

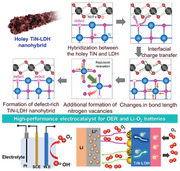

## 1 Introduction

The fine‐control of defect structure has evoked a great deal of research activity because of its usefulness in improving the diverse energy‐related functionalities of inorganic solids for application as electrocatalysts, electrodes, photocatalysts, and adsorbents.^[^ ^1^, ^2^, ^3^, ^4^ ^]^ The great impact of defect introduction in terms of optimizing the catalytic and electrochemical performances originates from the provision of surface active sites, the lowering of the reaction activation energy, an improvement in the mass transport kinetics, and the optimization of reactant/product adhesion.^[^ ^5^, ^6^ ^]^ Due to the remarkable advantages of defect control, several synthetic methodologies have been developed for the preparation of defective materials, such as thermal treatment, chemical reduction, photoreduction, selective etching, and plasma treatment.^[^ ^7^, ^8^ ^]^ However, most of these methods demand the application of harsh synthetic conditions like high temperature and strong acidic/alkaline media, and/or the use of energy‐consuming apparatus, which limit the universal use of these methods.^[^ ^9^, ^10^, ^11^ ^]^ Therefore, it is necessary to develop a new versatile and environmentally‐benign methodology to create crystal vacancies under mild condition without use of special apparatus. As an alternative synthetic strategy to explore high‐performance functional materials, hybridization between nanostructured species has attracted increasing research interest since the formation of a synergetic coupling between hybridized components enables not only an improvement in the pre‐existing properties of each component but also the creation of unexpected functionalities.^[^ ^12^, ^13^ ^]^ Based on the expectation that coordinatively‐unsaturated crystal defects can act as efficient binding sites to achieve strong interfacial electronic couplings with hybridized species, the use of a defective conductive species as a hybridization matrix is expected to be effective in maximizing the hybridization effect through the formation of interfacial coordinative bonding based on the inner sphere mechanism.^[^ ^14^, ^15^ ^]^ As a result of the enhanced interfacial charge transfer in the defective hybrid structure, the crystal vacancy can be further created by a significant change in the formation enthalpy of the defective hybrid structure. The resulting electronic‐coupling‐driven defect control is supposed to offer a powerful means to optimize diverse functionalities of inorganic materials via the synergetic combination of two beneficial design factors of defect engineering and interfacial electronic coupling. One of the most promising defective substrates for strongly‐coupled hybrids is holey inorganic nanotubes (NTs), which exhibit a high electrical conductivity, numerous crystal defects, and efficient mass diffusion pathways. The formation of nitrogen vacancies upon the aliovalent substitution of oxide (O^2−^) with nitride (N^3−^) renders holey TiN NTs a useful substrate for achieving strong interfacial chemical interaction with hybridized species.^[^ ^16^, ^17^, ^18^, ^19^ ^]^ Hybridization with conductive TiN NTs is expected to be effective in improving the electrical conductivities and electrochemical activities of poorly conductive materials, such as layered double hydroxides (LDHs). Notably, the presence of numerous surface‐exposed anions of ultrathin inorganic nanosheets (NSs) promotes the formation of interfacial coordination bonding with anion‐defective TiN NTs, in addition to the simultaneous stabilization of crystal vacancies. The resulting enhanced interfacial electronic coupling with defective conductive TiN matrix is supposed to enhance the redox capability of component ions in electrochemically‐active LDH NSs and thus to optimize the electrode and electrocatalyst performances of hybrid materials. Despite considerable research activities devoted to defect control in inorganic materials,^[^ ^20^, ^21^ ^]^ at the time of this submission, we are unaware of any other report relating to the remarkable mutual reinforcement between defect formation and interfacial electronic coupling in intimately‐coupled hybrid system and the resulting enhancement of redox activity.

Thus, we herein report a novel hybridization‐driven in situ defect formation and the accompanying reinforcement of interfacial electronic coupling and electrocatalytic redox cationic activity using anion‐defective holey TiN−LDH nanohybrids to provide a novel synthetic strategy toward high‐performance electrocatalysts. Evolutions of the crystal defects, the electronic structures, and the cation redox capability upon hybridization are systematically studied using a series of in situ spectroscopic and theoretical characterization techniques. The obtained defective TiN−LDH nanohybrids are tested as electrocatalysts for the oxygen evolution reaction (OER) and Li−O_2_ batteries to elucidate the crucial roles of crystal defects and interfacial electronic coupling in optimizing their electrochemical performances.

## 2 Results and Discussion

### 2.1 Synthesis of Holey TiN NT−LDH Nanohybrids with Controlled Defect Structures

**Figure 1:**
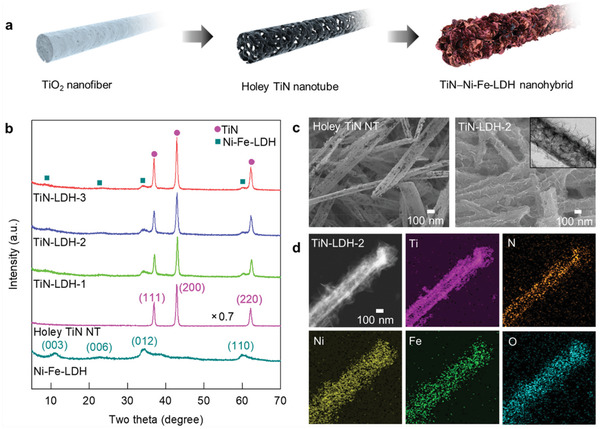
a) Schematic illustration for the synthesis of the TiN−LDH nanohybrid, b) powder XRD patterns, c) FE‐SEM and TEM (inset) images, and d) EDS−elemental mapping data.

As a hybridization matrix, holey TiN NTs were synthesized by the NH_3_ treatment of TiO_2_ nanofiber at 900 °C.^[^ ^16^ ^]^ Transmission electron microscopy (TEM) analysis clearly confirmed the formation of holey NTs (Figure S1, Supporting Information), indicating a significant morphological change from the nanofiber to holey NTs upon NH_3_ treatment. As presented in Figure S2 and Table S1, Supporting Information, the phase transformation from TiO_2_ to TiN upon ammonolysis was confirmed by Rietveld refinement analysis, which showed the formation of two closely‐related titanium nitride phases of TiN_0.88_ and TiN_0.9_ in a ratio of 72:28, thereby indicating the presence of nitrogen vacancies in the holey TiN NTs. The defective nature of this material was further evidenced by Ti K‐edge extended X‐ray absorption fine structure (EXAFS) fitting analysis, which indicated a smaller coordination number (CN) of the (Ti−N) coordination shell in the case of the holey TiN NTs compared to the non‐holey TiN (Figure S3, Supporting Information; **Table** **1**). The crystal growth of Ni−Fe–LDH NSs on the holey TiN NTs yields intimately‐coupled TiN−LDH nanohybrids with the TiN/LDH molar ratios of 1.6, 2.0, and 2.4 (denoted as TiN−LDH‐1, TiN−LDH‐2, and TiN−LDH‐3, respectively), see **Figure** **1**a. Hybridization between the TiN and LDH species was confirmed by powder X‐ray diffraction (XRD) observations, where the distinct Bragg reflections of the TiN and LDH phases for the TiN−LDH nanohybrids were observed; see Figure 1b. It was also observed that lowering of the LDH content gave rise to a gradual weakening of the (*00l*) reflections of the LDH component, indicating the depressed content and/or self‐stacking of LDH NSs via the homogeneous dispersion on the surface of the TiN NTs. Field emission‐scanning electron microscopy (FE‐SEM) and TEM analyses clearly demonstrate the anchoring of very thin LDH NSs on the surfaces of the holey TiN NTs (Figure 1c; Figure S4, Supporting Information), which differ significantly from the case of the heavily‐aggregated microflower morphology of the pristine LDH material (Figure S1, Supporting Information). Moreover, energy dispersive X‐ray spectroscopy (EDS)−elemental mapping analysis provided strong evidence for the homogeneous distribution of Ti, N, Ni, Fe, and O in the entire TiN−LDH‐2 nanohybrid (Figure 1d).

**Table 1:** Results of non‐linear least‐squares EXAFS fitting analysis for TiN−LDH nanohybrids

Material	Bonding pair	CN	R [Å]	*ΔE* [eV]	*σ* ^2^ [Å^2^]	F
Non‐holey TiN	(Ti−N)	6.00	2.093	0.95	0.0090	0.0019
Holey TiN NT	(Ti−N)	5.23	2.087	4.55	0.0084	0.0027
Ni−Fe–LDH	(Ni−O)	5.75	2.026	2.74	0.0056	0.0012
	(Fe−O)	5.72	1.977	5.14	0.0079	0.0081
TiN−LDH‐1	(Ti−N)	4.30	2.079	2.00	0.0075	0.0027
	(Ni−O)	5.77	2.035	1.39	0.0053	0.0100
	(Fe−O)	5.63	1.988	0.53	0.0061	0.0099
TiN−LDH‐2	(Ti−N)	4.46	2.071	1.89	0.0084	0.0036
	(Ni−O)	5.78	2.037	1.25	0.0057	0.0068
	(Fe−O)	5.72	1.987	2.84	0.0067	0.0110
TiN−LDH‐3	(Ti−N)	4.79	2.077	2.05	0.0081	0.0021
	(Ni−O)	5.81	2.037	1.83	0.0057	0.0079
	(Fe−O)	5.68	1.987	1.08	0.0059	0.0056
Non‐holey‐TiN−LDH‐2	(Ti−N)	5.82	2.091	0.80	0.0110	0.0039
	(Ni−O)	5.80	2.028	0.58	0.0050	0.0021
	(Fe−O)	5.82	1.980	0.94	0.0073	0.0095

### 2.2 Interfacial Electronic Coupling and Hybridization‐Driven Defect Formation

**Figure 2:**
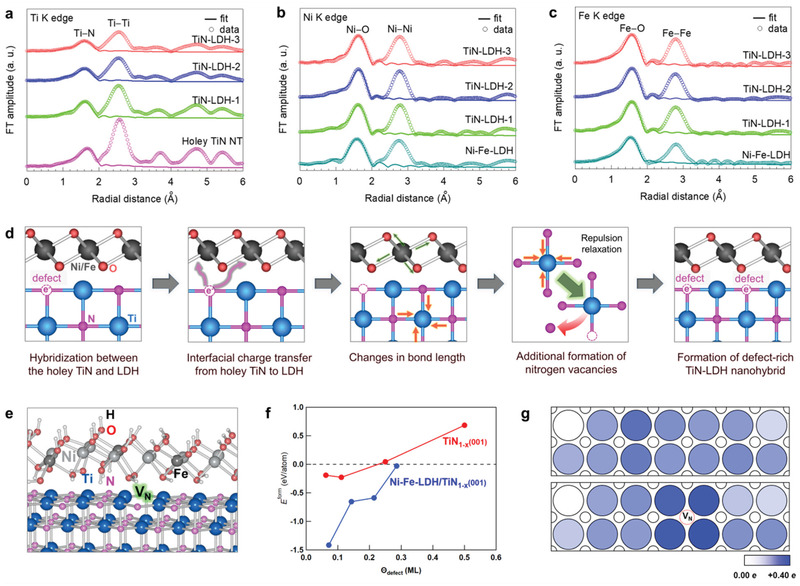
a) Ti K‐edge FT EXAFS data, b) Ni K‐edge FT EXAFS data, c) Fe K‐edge FT EXAFS data, d) schematic model for hybridization‐driven defect formation on TiN NTs, e) DFT‐optimized interface structure of Ni−Fe‐LDH/TiN (with and without nitrogen vacancies), f) nitrogen vacancy formation energy at the interface as a function of *Θ*_defect_ (for the definition of *Θ*_defect_, see the text) under nitrogen lean conditions, and g) degree of charge depletion of interfacial Ti atoms (large circles) in Ni−Fe‐LDH/TiN (with and without nitrogen vacancies) taken with respect to that of TiN(001).

The interfacial electronic coupling and hybridization‐induced defect formation in the TiN−LDH nanohybrids were systematically investigated using a series of spectroscopic techniques. According to the results of Ti K‐edge EXAFS (**Figure** **2**a; Figure S5, Supporting Information), all TiN−LDH nanohybrids commonly displayed nearly identical Fourier transformed (FT) EXAFS spectra to that of the holey TiN, confirming the maintenance of the rocksalt‐structured TiN phase upon hybridization. As listed in Table 1, hybridization with Ni−Fe–LDH shortens the (Ti−N) bond distance of the holey TiN NTs, indicating an increase in the Ti oxidation state. Since the lattice enthalpy of TiN is inversely proportional to the (Ti−N) bond distance, the observed bond shortening implies an increase in the lattice enthalpy of the defective TiN, which can compensate for loss of lattice energy caused by the formation of nitrogen vacancies. Such a structural modification upon hybridization leads to the stabilization of the defective TiN structure, resulting in the additional formation of crystal vacancies. In fact, the EXAFS fitting analysis clearly demonstrates that the hybridization of holey TiN NTs with Ni−Fe–LDH NSs causes a distinct lowering of the CN for the (Ti−N) coordination shell (Table 1), thereby confirming the creation of nitrogen vacancies caused by the electronic coupling with LDH.^[^ ^22^ ^]^ The present EXAFS results provide strong evidence for the charge‐transfer‐driven defect formation of TiN NTs assisted by accompanying increases in the lattice energy and entropy, as illustrated in Figure 2d. Moreover, the additional formation of nitrogen defect upon the hybridization was cross‐confirmed by micro‐Raman and scanning transmission electron microscopy (STEM) analyses for TiN−LDH‐2. Since most of the TiN surfaces are covered with LDH NS crystallites, micro‐Raman and STEM data were measured from the edge sites of exposed TiN components. As shown in Figure S6, Supporting Information, hybridization with LDH gives rise to a distinct blue shift of Raman peak at ≈320 cm^−1^, which is assigned as the acoustic mode of TiN. Since the energy of this phonon line is proportional to the concentration of nitrogen vacancies,^[^ ^23^ ^]^ the observed blue‐shift of this Raman peak upon hybridization provides clear evidence for the additional creation of nitrogen defects. In addition, the creation of nitrogen defect upon the hybridization was further supported by the STEM analysis exhibiting the shorter Ti−Ti bond distance for TiN−LDH than TiN (Figure S7, Supporting Information). Although very low electron density of nitrogen prevents from directly probing this light element by STEM technique, the decrease of (Ti−Ti) bond distance upon the hybridization can be interpreted as indirect proof for the formation of nitrogen vacancies, which causes the reinforcement of adjacent (Ti−Ti) bonds. As plotted in Figure 2b,c, the Ni K‐edge and Fe K‐edge EXAFS spectra of the TiN−LDH nanohybrids appear comparable to those of the pristine Ni−Fe–LDH, verifying the maintenance of the LDH lattice upon hybridization. In addition, the curve fitting analysis clearly demonstrates that the TiN−LDH nanohybrids have somewhat longer (Ni−O) and (Fe−O) bond distances than those of the pristine Ni−Fe–LDH (Table 1), indicating a significant reduction in the Ni/Fe oxidation state due to the interfacial electron transfer from holey TiN. In contrast to (Ti−N) bond, the (Ni−O) and (Fe−O) bonds of the TiN−LDH nanohybrids have similar CNs to those of the pristine LDH, reflecting the negligible formation of crystal defects in the LDH component (Table 1). Since the observed elongation of the (Ni/Fe−O) bond distances implies a decrease in the lattice energy of the LDH, the interfacial charge transfer cannot induce the formation of crystal vacancies in the LDH components. This is in stark contrast to the enhanced defect formation observed in the TiN component.

The evolution of the chemical bonding nature of the bulk and surface species upon hybridization was separately investigated comparatively using a combination of bulk‐sensitive X‐ray absorption near‐edge structure (XANES) and surface‐sensitive X‐ray photoelectron spectroscopic (XPS) analyses. As plotted in Figure S8, Supporting Information, all TiN−LDH nanohybrids displayed typical Ti K‐edge XANES spectral features corresponding to the TiN phase but with a slightly higher edge energy than that of the holey TiN NTs, confirming a slight increase in the trivalent oxidation state of the bulk Ti species upon hybridization with LDH. In contrast to the Ti K‐edge XANES results, which show typical TiN‐like features, the Ti 2p XPS data for the TiN−LDH nanohybrids and the holey TiN NTs display broad features attributed to Ti^3+^ and Ti^4+^ components, thereby indicating the presence of highly oxidized Ti^4+^ species on the surface as well as Ti^3+^ species in the bulk (Figure S9, Supporting Information). In addition, hybridization with LDH gives rise to a notable increase in the Ti^4+^/Ti^3+^ ratio in the XPS data, highlighting that oxidation of the Ti species took place in the interface/surface region due to the interfacial electron transfer from TiN to Ni−Fe–LDH. This spectral modification observed upon hybridization with LDH is more prominent in the Ti 2p XPS data than in the Ti K‐edge XANES spectra, therefore clarifying the stronger hybridization effect on the interface/surface species compared to on the bulk species. It is worth mentioning that the binding energy (BE) of the Ti^4+^ component decreases after coupling with LDH. Considering that the creation of nitrogen vacancies in TiN would cause reinforcement of the remaining (Ti−N) bonds with an enhanced bond covalency, the observed lower energy shift of the interface/surface Ti^4+^ species upon hybridization with LDH can be interpreted as further evidence for the increase in the nitrogen defect content and the resulting enhancement of the (Ti−N) bond covalency in the interfacial region. The occurrence of distinct interfacial charge transfer from TiN to Ni−Fe–LDH was further confirmed by the lower BEs of the Ni 2p and Fe 2p XPS peaks for the TiN−LDH nanohybrids compared to those of the pristine Ni−Fe–LDH, indicating a decrease in the Ni and Fe oxidation states upon hybridization (Figure S9, Supporting Information). Again, the alteration of the edge energies in the Ni K‐edge and Fe K‐edge XANES spectra was less prominent than the peak shift in the Ni 2p and Fe 2p XPS data, confirming the stronger hybridization effect on the interface region than on the bulk region.

According to the N_2_ adsorption−desorption isotherm measurements (Figure S10, Supporting Information), all TiN−LDH nanohybrids exhibited more prominent adsorption of N_2_ molecules than the pristine Ni−Fe–LDH, underscoring the remarkable increase in porosity upon hybridization. The increase in the surface area of Ni−Fe‐LDH upon hybridization was therefore confirmed by surface area calculations based on the Brunauer−Emmett−Teller (BET) equation. All nanohybrids were found to possess expanded surface areas of 220, 203, and 185 m^2^ g^−1^, that is, TiN−LDH‐1, TiN−LDH‐2, and TiN−LDH‐3, respectively; these values are significantly larger than those of Ni−Fe–LDH (163 m^2^ g^−1^) and the holey TiN NTs (19 m^2^ g^−1^). This remarkable surface expansion upon hybridization was attributed to the effective dispersion of thin LDH NSs on the TiN NT surfaces, and/or to the hybridization‐induced defect formation in TiN NTs.^[^ ^24^ ^]^

### 2.3 Effect of Electronic Coupling on Defect Formation Energetics

To theoretically investigate the effect of hybridization on the defect structure and the simultaneous evolution of interfacial electronic coupling, Bader charge analysis was performed for the Ni−Fe–LDH/TiN interface structures with nitrogen vacancies. Figure 2e illustrates the density functional theory (DFT)‐optimized structural model of Ni_0.75_Fe_0.25_(OH)_2_ on TiN(001) with a surface nitrogen vacancy coverage (*Θ*_defect_) of ≈0.07 monolayers (MLs), in which defect is defined as the ratio of the number of surface nitrogen vacancies to the total number of nitrogen atoms in the outermost layer for defect‐free pristine TiN(001). The surface nitrogen vacancies with different defect values ranging from 0.07 to 0.29 MLs were introduced at the outermost surface layer of TiN, which is in contact with the LDH layer. As plotted in Figure 2f, we calculated the formation energy (under the nitrogen lean condition using Equation (1)) as a function of defect to determine the thermodynamic favorability of surface nitrogen vacancy formation at the interface. Our results demonstrate that the interface nitrogen vacancy at a low defect below 0.29 MLs is thermodynamically favorable for the TiN−LDH nanohybrid. Of prime importance is that the surface nitrogen vacancies formed at the interface of TiN−LDH are thermodynamically more favorable than those on TiN(001), providing strong theoretical support for the hybridization‐driven formation of nitrogen vacancies. However, for *Θ*_defect_ > 0.30 MLs, the interface nitrogen vacancies are deemed thermodynamically unfavorable, strongly suggesting that the repulsive interaction of nitrogen defects in holey TiN NTs prevent the local clustering of nitrogen defects.

The effect of defect introduction on the interfacial electronic coupling was then studied theoretically by calculating the amount of electronic charge transfer in the TiN−LDH nanohybrid system with (at *Θ*_defect_ = 0.07 MLs) and without nitrogen vacancies at the interface. The changes in the effective Bader charges (*Δq*_eff_) of the TiN−LDH nanohybrid were calculated from the effective Bader charges (*q*_eff_) of the LDH and TiN components. Here, *q*_eff_ is taken as the numerical difference between the number of valence electrons and the integrated Bader charges within a Bader volume of the atom.^[^ ^25^ ^]^ As tabulated in Table 2; Table S2, Supporting Information, the averaged *Δq*_eff_ values of the Ti atoms in TiN−LDH with and without nitrogen vacancies were determined to be +0.10 and +0.09 e, respectively, thereby confirming the occurrence of charge depletion in the outermost layer of TiN. These electrons from the TiN layer (mainly Ti and partially N atoms) are transferred to mostly Ni (−0.18 and −0.21 e) and partially Fe atoms (−0.05 and −0.05 e) in the LDH layers, resulting in a reduction of the formal charge states of the Ni and Fe atoms, although the charge transfer process was subtly different for the cases with and without nitrogen vacancies. While the overall depletion of charges in the TiN layer is relatively homogeneous in the absence of nitrogen vacancies, the presence of nitrogen defects enhances a localized charge transfer near the interface nitrogen vacancy, as depicted in Figure 2g. This “localized electron pocket” has been previously proposed to act as an effective chemical anchoring site for foreign material.^[^ ^26^ ^]^ This calculation result emphasizes that the use of defective TiN NTs is more effective in forming a strongly‐coupled hybrid material with enhanced charge transfer properties than in the case of a non‐defective TiN material. In addition, our DFT‐calculated Bader charge analysis clearly demonstrates a robust electronic coupling in the TiN−LDH nanohybrid, which is in good agreement with the EXAFS results (Figure 2a−c). To quantify the interfacial electronic coupling, the changes in the net and local magnetic moments for these TiN−LDH nanohybrids were inspected upon the introduction of interface nitrogen defects. As illustrated in Figure S11, Supporting Information, in line with our Bader charge analysis, excess electrons accumulate mostly on the Ni atoms (and partially on the Fe atoms), resulting in a reduction of local magnetic moments (M in *μ*_B_) for the Ni and Fe atoms (Figure S12, Supporting Information). This correlation between the degree of charge transfer from TiN and the magnetization of the Ni and Fe atoms in LDH unequivocally lends strong theoretical support to the enhanced electronic coupling of the LDH and TiN upon the introduction of nitrogen vacancies.

### 2.4 OER Electrocatalyst Functionalities and Li−O_2_ Electrode Performances

**Figure 3:**
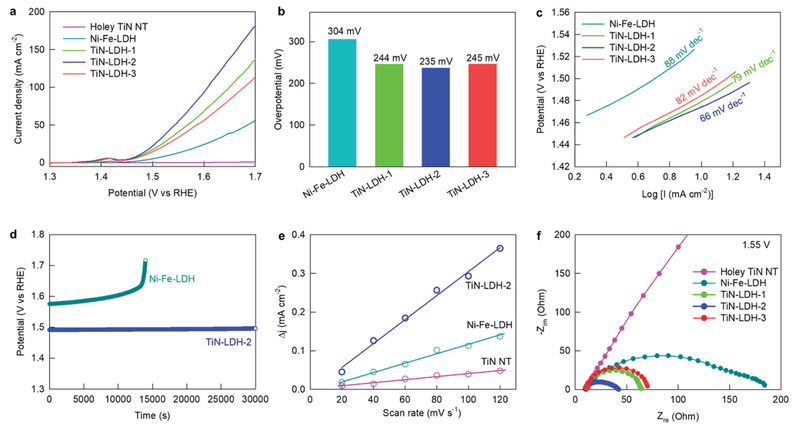
a) LSV curves for OER, b) overpotentials at 10 mA cm^−2^, c) Tafel plots, d) chronopotentiometric curve recorded at 10 mA cm^−2^, e) plots of current density difference versus scan rate, and f) EIS data.

The present TiN−LDH nanohybrids were then employed as OER electrocatalysts to investigate the influences of defect introduction and the enhanced interfacial electronic coupling on their energy‐related performances. As shown in the linear sweep voltammetry (LSV) data presented in **Figure** **3**a, the holey TiN NTs are poor OER catalysts, failing to reach a current density of 10 mA cm^−2^, whereas a significant OER activity was detected for the pristine Ni−Fe–LDH, with an overpotential of 304 mV at 10 mA cm^−2^. As shown in Figure 3b, the TiN−LDH nanohybrids exhibit significantly higher OER electrocatalytic activities with lower overpotentials and higher current densities than the pristine Ni−Fe–LDH and holey TiN NTs, highlighting the remarkable advantage of intimate hybridization with defective TiN. Among the nanohybrids presented herein, the best OER performance with an overpotential of 235 mV was obtained for TiN−LDH‐2, which contained an intermediate TiN/LDH ratio; this was the result of a compromise between the positive effect of hybridization with conductive TiN and the negative effect of the lowering of the electrocatalytically‐active LDH content.

The crucial role of nanoscale hybridization in improving the electrocatalyst performance was confirmed by the significantly lower OER activity of the physical mixture of holey TiN NTs and Ni−Fe‐LDH compared to that of TiN−LDH‐2 (Figure S13, Supporting Information). As plotted in Figure 3c, the TiN−LDH nanohybrids commonly demonstrate lower Tafel slopes of 66−82 mV dec^−1^ compared to that of Ni−Fe‐LDH (88 mV dec^−1^), indicating an improvement in the OER kinetics upon hybridization with TiN.^[^ ^27^ ^]^ In addition, as presented in Figure 3d, TiN−LDH‐2 shows a more stable OER activity than the LDH material at a current density of 10 mA cm^−2^, underscoring the beneficial effect of hybridization. Moreover, powder XRD and FE‐SEM analyses exhibited no marked chages in crystal structure and morphology after the stability test (Figure S14, Supporting Information). These results can be regarded as strong evidence for the maintenance of original crystal structure after the catalytic reaction, confirming the excellent structural stability of TiN−LDH‐2. As can be seen from Figure 3e, the electrochemical surface area (ECSA) of TiN−LDH‐2 was calculated to be 1.55 mF cm^−2^ from the electrochemical double‐layer capacitance, and this value is significantly larger than those of the pristine LDH (0.56 mF cm^−2^) and the holey TiN NTs (0.17 mF cm^−2^). The observed increase in the ECSA upon hybridization was attributed to the morphological change of Ni−Fe–LDH to give single‐/few‐layered NSs with optimal microporosities, as well as to the creation of hybridization‐induced defects. According to electrochemical impedance spectroscopy (EIS) analysis (Figure 3f), the hybridization of Ni−Fe‐LDH with TiN significantly reduces the diameter of the semicircle at 1.55 V versus the reversible hydrogen electrode (RHE), highlighting an enhancement in the charge transfer kinetics.^[^ ^28^ ^]^ Among the various nanohybrids under investigation, TiN−LDH‐2 exhibited the lowest charge transfer resistance (R_ct_), and the obtained value was significantly smaller than those of pristine Ni−Fe–LDH and the holey TiN NTs.^[^ ^29^ ^]^ Such an improvement in the charge transfer kinetics makes a significant contribution to the beneficial effect of hybridization with holey TiN NTs on the electrocatalytic performance of the LDH.

**Figure 4:**
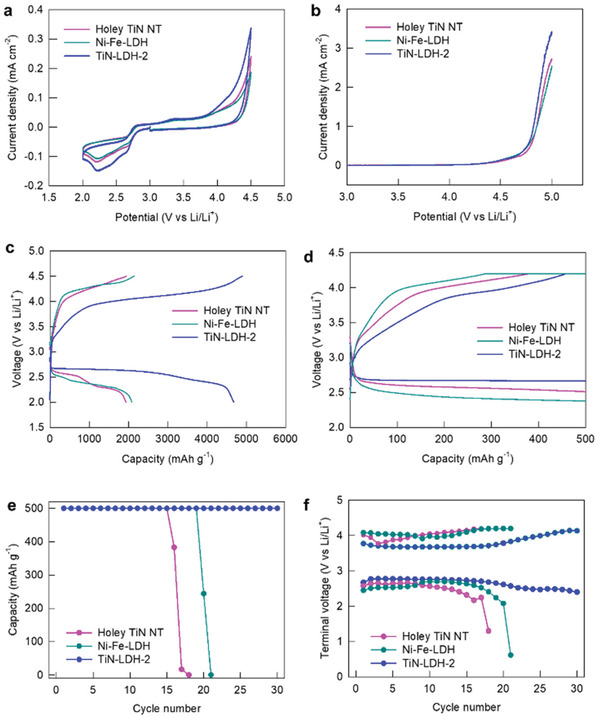
a) CV, b) LSV, c) full‐range discharge–charge curves, d) limited capacity discharge−charge curves, e) capacity cycle performance, and f) cycle terminal voltage behaviour.

Based on the excellent electrocatalytic performance of TiN−LDH‐2, this nanohybrid was employed as a cathode catalyst for Li−O_2_ batteries to verify the universal effectiveness of the present synthetic strategy. According to cyclic voltammetry (CV) analysis, TiN−LDH‐2 exhibits a more intense cathodic peak at ≈2.25 V and a better stability at a higher oxidation potential compared to those of the TiN NTs and Ni−Fe‐LDH, see **Figure** **4**a,b. As plotted in the galvanostatic charge−discharge profiles of the Li−O_2_ cells (Figure 4c), the TiN−LDH‐2 nanohybrid delivered a much larger capacity of 4680 mAh g^−1^ with a lower overpotential of 1.65 V compared to the corresponding values for the holey TiN NTs (1932 mAh g^−1^ and 1.77 V) and Ni−Fe–LDH (2083 mAh g^−1^ and 1.99 V), emphasizing the beneficial role of hybridization on the performance of Li−O_2_ batteries. This improvement in the electrode functionality of the nanohybrid sample can be ascribed to the effective anchoring of the LDH NSs on the holey TiN NT substrate via the interfacial electronic coupling that effectively exposes the catalytically active sites, securing larger ECSA values for TiN−LDH‐2. Additionally, the 2D sheets on 1D NTs provide a 3D framework with open channels for fast reactants/electrolyte diffusion and increased electronic conductivity, a significant advantage for the hybrid TiN−LDH‐2 which also contributes to its high BET surface area.^[^ ^30^, ^31^, ^32^ ^]^ Given that BET and ECSA are directly related to the catalytic activity, the improved capacity obtained for TiN−LDH‐2 compared to TiN NT and LDH is reasonable.

To examine the kinetic behaviors of the present materials during multiple cycling, the Li−O_2_ cells were tested with a limited charge−discharge capacity of 500 mAh g^−1^. As shown in the first charge and discharge curves in Figure 4d, TiN−LDH‐2 exhibits a lower overpotential (1.52 V) than the holey TiN NTs (1.71 V) and Ni−Fe–LDH (1.68 V), confirming the positive effect of hybridization on the cathode performance. The charge transfer across the interface relaxes the inherent Fe−O bond, making the Fe susceptible to moderate interactions with superoxide ions (O_2_^−^) to facilitate discharging/charging process at low activation energies, leading to lower overpotentials. Resultantly, TiN−LDH‐2 exhibited a stable reversible cycle retention up to the 30th cycle without fading (Figure 4e), whereas an abrupt decay in capacity occurred for both the holey TiN NTs and Ni−Fe–LDH prior to the 20th cycle. As shown in the terminal voltage profiles during cycling (Figure 4f), both the charge and discharge terminal voltages of TiN−LDH‐2 were maintained within the set voltage cut‐off, which is in stark contrast to the holey TiN NTs and Ni−Fe–LDH. The obtained electrochemical data therefore highlight the superiority of hybridization with holey TiN NTs on the Li−O_2_ electrode functionality of LDH.

### 2.5 Effect of Holey Morphology on Interfacial Electronic Coupling and Functionality

**Figure 5:**
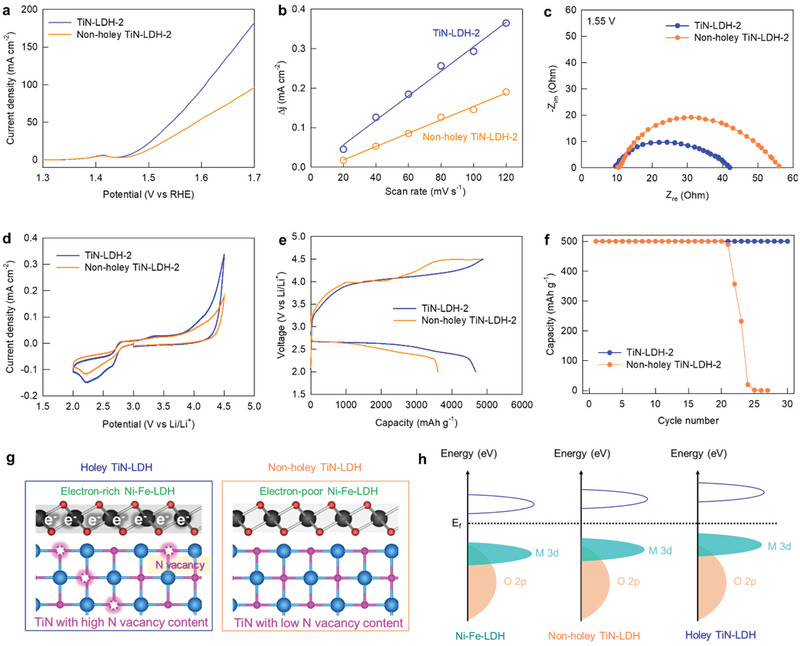
a) LSV, b) ECSAs data, c) EIS, d) CV, e) full‐range discharge−charge curves, f) capacity cycle performance, g) schematic model of TiN−LDH hybrid, and h) schematic rigid band diagrams.

To verify the unique advantage of the holey NT morphology with nitrogen vacancies in enhancing the hybridization effect, an additional nanohybrid of Ni−Fe‐LDH was prepared as a reference using non‐holey TiN with a TiN/LDH molar ratio of 2.0 (denoted as non‐holey‐TiN−LDH‐2). The intimate hybridization between the non‐holey TiN particles and Ni−Fe–LDH was confirmed by powder XRD and FE‐SEM analyses, which demonstrated the homogeneous immobilization of Ni−Fe–LDH NSs on the surfaces of the non‐holey TiN particles, whose dimensions are comparable to those of the holey TiN NTs, as shown in Figures S15 and S16, Supporting Information. According to the N_2_ adsorption−desorption isotherms (Figure S17, Supporting Information), a smaller surface area (114 m^2^ g^−1^) was found for the non‐holey‐TiN−LDH‐2 nanohybrid compared to the holey‐TiN−LDH‐2 (203 m^2^ g^−1^), indicating the inferior role of non‐holey TiN in increasing the porosity of the nanohybrid. As listed in Table 1, Ti K‐edge EXAFS fitting revealed that the non‐holey‐TiN−LDH‐2 nanohybrid exhibited a significantly weaker decrease in CN and weaker bond shortening for the Ti−N coordination shell compared to the holey‐TiN−LDH‐2 nanohybrid. Similarly, as compared with the holey‐TiN−LDH‐2, the non‐holey‐TiN−LDH‐2 nanohybrid underwent less eminent changes in the Ni K‐edge and Fe K‐edge EXAFS fitting results upon hybridization (Figure S18, Supporting Information; Table 1), underscoring the critical role of the TiN nitrogen vacancies in enhancing the interfacial electronic coupling with LDH. The advantage of the holey conductive substrate with anion defects in optimizing the energy‐related performance of the nanohybrid was evident upon comparison of the OER electrocatalytic activity and the Li−O_2_ electrode performance of the non‐holey‐TiN−LDH‐2 with that of the holey‐TiN−LDH‐2. As shown in **Figure** **5**a, the non‐holey‐TiN−LDH‐2 displayed an inferior OER performance (overpotential of 263 mV) compared to the holey‐TiN−LDH‐2 (235 mV), emphasizing the crucial role of the holey NTs morphology in optimizing the electrocatalytic activity of the TiN−LDH nanohybrid. The smaller ECSA of the non‐holey TiN−LDH‐2 compared to the holey‐TiN−LDH‐2 verifies the importance of the holey NT morphology in enhancing the exposure of active sites to the electrolyte (Figure 5b). According to the EIS analysis, the lowering of R_ct_ upon hybridization is less prominent for the non‐holey‐TiN−LDH‐2 nanohybrid compared to the holey‐TiN−LDH‐2 nanohybrid, thereby indicating the remarkable advantage of vacancy introduction and the resulting enhanced interfacial electronic coupling in improving the transport properties of the nanohybrid (Figure 5c). As presented in Figure 5d,e, the non‐holey‐TiN−LDH‐2 nanohybrid displayed an inferior Li−O_2_ battery performance with smaller capacity (3610 mAh g^−1^) compared to the holey TiN−LDH‐2. Of prime importance is the fact that the cyclability of the non‐holey‐TiN−LDH‐2 nanohybrid was also found to be inferior to that of the holey TiN−LDH‐2 (Figure 5f), thereby confirming the crucial role of the holey NT morphology in optimizing the Li−O_2_ electrode performance. This observation is attributable to the depressed electronic coupling and negligible defect introduction upon the hybridization of non‐holey TiN with LDH, which ultimately leads to an insignificant improvement in the mass transport properties and the charge transfer kinetics. Moreover, the relatively electron‐rich surface of LDH caused by the stronger electron transfer from holey TiN in holey TiN−LDH‐2 than in non‐holey TiN−LDH‐2 raised d band center of LDH (Figure 5g,h). This d‐band shift results in an enhancement in the binding strength toward OH^−^ during OER process and thus improving the electrocatalytic activity of LDH material.^[^ ^33^, ^34^ ^]^ The defect‐enhanced interfacial interaction between the conductive TiN NTs and the electrochemically‐active LDH NSs in addition to the feedback formation of crystal vacancies can significantly contribute to the improved electrocatalyst/electrode functionalities of the holey TiN−LDH nanohybrid.

### 2.6 In Situ Spectroscopic Analysis During OER Activity Test

**Figure 6:**
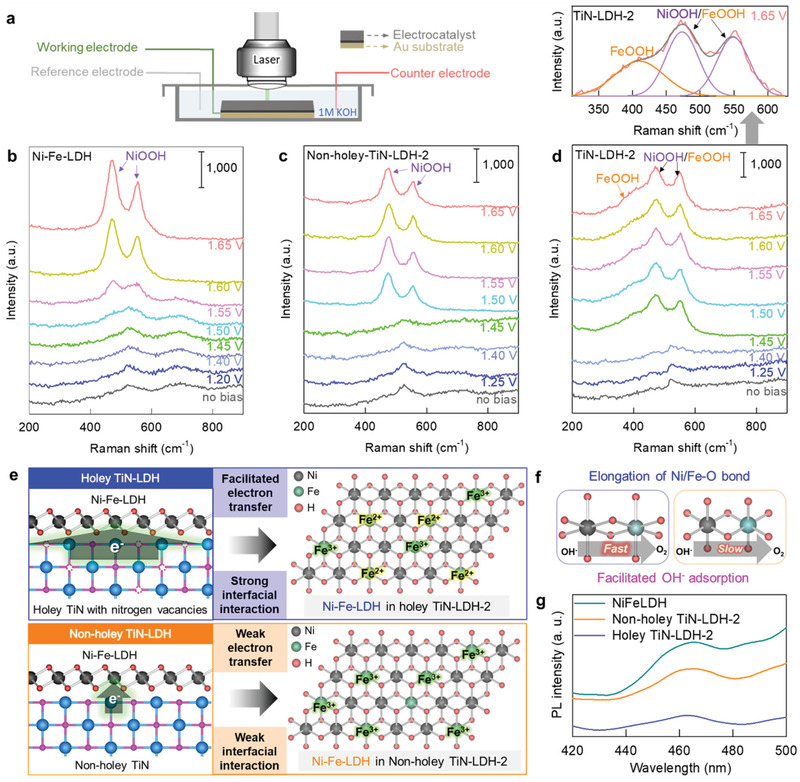
a) Schematic illustration of in situ SERS during the OER, b–d) in situ SERS data during the OER process, e,f) schematic model for holey TiN−LDH and non‐holey TiN−LDH, and g) PL data.

To accurately understand the electrocatalytic reaction mechanism, evolutions of the structural changes of the present electrocatalysts during OER process were investigated with in situ surface‐enhanced Raman spectroscopy (SERS) analysis (**Figure** **6**a). As shown in Figures 6b−d, all the catalyst materials without bias commonly display typical phonon lines of Ni−Fe‐LDH showing the Raman peaks at ≈530 and ≈690 cm^−1^.^[^ ^35^ ^]^ While a gradual increase of potential during OER process induces the disappearance of Ni−Fe‐LDH phonon lines, strong Raman signals at ≈480 and ≈560 cm^−1^ corresponding to the *γ*‐NiOOH^[^ ^36^, ^37^ ^]^ appeared at 1.55 V for pristine Ni−Fe‐LDH, 1.50 V for non‐holey TiN−LDH‐2, and 1.45 V for TiN−LDH‐2, respectively. This observation confirms the transformation of Ni(OH)_2_ to NiOOH on the surface of Ni−Fe–LDH and the enhanced electrocatalytic activity of Ni−Fe‐LDH upon the hybridization with TiN. It is worth noting that the holey TiN−LDH‐2 displays additional Raman peak corresponding to the FeOOH phase at ≈410 cm^−1^ during OER process as well as the NiOOH‐related phonon lines, as plotted in Figure 6d (top).^[^ ^38^ ^]^ This is in stark contrast to non‐holey TiN−LDH‐2 and pristine Ni−Fe‐LDH showing no FeOOH‐related phonon lines. The co‐formation of FeOOH and NiOOH phases in holey TiN−LDH‐2 during OER provides strong evidence for the participation of lattice Fe ions as well as Ni ions of the Ni−Fe‐LDH component in the catalytic OER process, highlighting the maximization of the cation redox activity of LDH phase. This is the first spectroscopic evidence for the effective electrocatalytic activation of all the redoxable metal components of the LDH phase, which is quite effective in enhancing its electrocatalytic activity. Since the hybridization between holey TiN and Ni−Fe–LDH causes strong interfacial charge transfer from holey TiN to Ni−Fe‐LDH, the high electron density distributions of Ni−Fe–LDH lead to the partial reduction of Fe^3+^ to Fe^2+^ and elongation of (Fe−O) bond (Figure 6e,f). The weakening of (Fe−O) bond distance upon hybridization with holey TiN NTs provides appropriate strength of interaction with OH^−^, which is beneficial for electrochemically activating the Fe ions and improving electrocatalyst activity for OER.^[^ ^39^ ^]^ The present in situ SERS spectroscopic results indicate holey TiN NTs with nitrogen vacancies can act as efficient hybridization matrix for electrocatalyst material with strong interfacial interaction and facial charge transfer, therefore verifying the usefulness of the hybridization strategy for controlling the defect structure of inorganic solids and enhancing their electrocatalytic redox activity. The strong interfacial interaction of LDH and holey TiN was further confirmed by the photoluminescence (PL) results, whereby a weaker PL intensity was observed for the holey‐TiN−LDH‐2 compared to the non‐holey‐TiN−LDH‐2, as shown in Figure 6g. Since the spectral weight of the PL signal provides a sensitive measure for the efficiency of electron−hole recombination,^[^ ^20^ ^]^ the enhanced depression of the PL peak upon hybridization with the holey TiN NTs provides clear evidence for the more efficient electronic coupling of LDH with defective conductive TiN NTs.

There are several contributing factors to the improvement of the cationic redox activity and electrochemical functionalities of LDH phase upon hybridization with holey TiN NT as follows; (1) As determined from the EXAFS and DFT data, the hybridization‐driven defect formation of TiN−LDH with numerous nitrogen vacancies produces more catalytically active sites and also improves the accessibility of the reactant OH^−^/O_2_ species to the electrocatalytically‐active sites of LDH.^[^ ^40^, ^41^ ^]^ The increase defect content of TiN−LDH also facilitates the fast transport of electrolyte in electrocatalytic reactions.^[^ ^42^, ^43^ ^]^ (2) The non‐agglomerated dispersion of thin LDH NSs on the strongly‐coupled TiN NT provides many effective anchoring sites for discharged product Li_2_O_2_, which contributes to the improved Li−O_2_ battery performance and cyclability.^[^ ^44^ ^]^ (3) An efficient interfacial electron transfer from TiN to Ni−Fe‐LDH results in a significant reduction of the Ni/Fe oxidation states with the elongation of the Ni/Fe−O bond distances. The increase in the Ni/Fe−O bond distances can facilitate the attachment of reactant OH^−^ species onto the LDH component, which is beneficial for promoting the occupation of both Fe and Ni ions of hybridized LDH species in the OER process.^[^ ^39^ ^]^ (4) As evidenced by EIS analysis, an efficient electronic coupling with conductive TiN NTs enhances the electrical conductivity and charge transfer kinetics of Ni−Fe‐LDH, which is also responsible for the improved electrochemical performance and redox activity of TiN−LDH.

## 3 Conclusion

The present study underscores that hybridization‐driven defect control using a defective conductive substrate provides an effective synthetic methodology to explore high‐performance electrocatalysts via the maximization of their cation redox activity, which relies on the mutual reinforcement between interfacial electronic coupling and crystal defect formation. The systematic spectroscopic and DFT analyses of the defective TiN−LDH nanohybrid clearly demonstrate that the coordinatively‐unsaturated Ti sites in the holey TiN NTs play important roles in maximizing the interfacial electronic coupling with LDH NSs. The resulting explicit internal charge transfer leads to feedback stabilization of the nitrogen vacancies in the TiN NTs, which can be ascribed to the increase in lattice energy caused by shortening of the Ti−N bond distance upon interfacial charge transfer. That is, in case of single‐component material, the formation of lattice defect driven by the increase in entropy is hindered by accompanying decrease in enthalpy due to the loss of chemical bonds, as supposed from the equation of Gibbs free energy change, *ΔG* = *ΔH* − *TΔS* (*H*: enthalpy, *S*: entropy, *T*: temperature). In case of strongly‐coupled hybrid material, the effective formation of interfacial coordination bondings between the hybridized components offers additional enthalpy gain, which compensates partly the loss of enthalpy caused by the formation of crystal vacancy. This results in the shift of equilibrium position toward a richer defect state (Figure S19, Supporting Information). Additionally, the elastic deformation of crystal lattice upon the strong interfacial interaction also contributes to the increase of crystal vacancy upon hybridization. The hybridization‐driven defect formation can provide an effective means of controlling the level of crystal vacancies, because the concentration of electronic‐coupling‐driven vacancy can be tuned by altering the degree of interfacial charge transfer between hybridized components via suitable alignment of their band alignments. The resulting synergetic combination of defect engineering with interfacial electronic coupling between hybridized species can offer a valuable synthetic strategy to optimize diverse functionalities of inorganic materials. Considering that the defect structure of many holey nanostructures can be tuned by the fine control of the heating conditions and atmosphere, in addition to anion substitution, the diverse electrochemical and photochemical functionalities of the holey defective nanohybrids could be further enhanced by optimizing of the defect structure and the resulting interfacial electronic coupling. Currently, we are investigating high‐performance photocatalyst materials prepared via the anchoring of semiconductor photocatalysts on the surfaces of holey conductive nanostructures with tailored defect structures.

## 4 Experimental Section

### Sample Preparation

The holey TiN NT was synthesized by high‐temperature heat‐treatment on TiO_2_ nanofibers under NH_3_ gas flow.^[^ ^16^ ^]^ As a precursor for holey TiN NT, TiO_2_ nanofiber was prepared by hydrothermal treatment of P25 powder in 10 m NaOH aqueous solution at 200 °C for 48 h. The obtained TiO_2_ nanofiber was subsequently sintered at 900 °C for 1 h with three different ramp rates (5 °C min^−1^ from 25 °C to 300 °C, 2 °C min^−1^ from 300 °C to 700 °C, and 1 °C min^−1^ from 700 °C to 900 °C) under NH_3_ flow (100 mL min^−1^), resulting in the formation of holey TiN NT. To promote the complete replacement of O^2−^ with N^3−^ with the minimization of structural frustration, three heating processes with different rates were employed. For hybridization between holey TiN NTs and Ni−Fe‐LDH NSs, 0.3 mmol of Ni(NO_3_)_2_⋅6H_2_O, 0.1 mmol of Fe(NO_3_)_3_⋅9H_2_O, 0.75 mmol of urea, and 0.05 mmol of Na_3_C_6_H_5_O_7_⋅2H_2_O were dissolved into the 50 mL aqueous suspension of holey TiN NTs. The mixture was stirred for 15 min, transferred to Teflon‐lined autoclave, and subjected to the hydrothermal‐treatment at 150 °C for 24 h. After the reaction, the product was obtained by centrifugation, washed with distilled water and ethanol thoroughly, and dried at 50 °C for 12 h. To examine the effect of composition on the properties of the resulting nanohybrids, several TiN/LDH molar ratios of 1.6, 2.0, and 2.4 were employed. As references, the Ni−Fe‐LDH material was obtained by the same synthetic process in the absence of TiN species whereas non‐holey‐TiN−LDH‐2 material was synthesized by the same synthetic process with non‐holey TiN.

### Sample Characterization

The crystal structures of the present materials were investigated by powder XRD using a Rigaku diffractometer with Ni‐filtered Cu K*α* radiation (Rigaku D/Max‐2000/PC, *λ* = 1.5418 Å, 298 K). The FE‐SEM, TEM, and STEM analyses were performed with Jeol JSM‐6700, Jeol JEM‐F200, and Jeol JEM‐ARM200F electron microscopes, respectively, to study the morphological properties of the present materials. The EDS−elemental maps were recorded with an energy‐dispersive X‐ray spectrometer equipped in the TEM machine. N_2_ adsorption−desorption isotherms of the present materials were measured at 77 K using Micromeritics ASAP 2020 to probe their pore structures and surface areas. Ti K‐edge, Ni K‐edge, and Fe K‐edge XANES/EXAFS analyses were carried out at beam line 10C of the Pohang Accelerator Laboratory (PAL, Pohang, Korea). The energy calibration for the collected XANES/EXAFS spectra was done by simultaneously measuring the reference spectrum of Ti, Ni, and Fe metal foil, respectively. The EXAFS analysis for the experimental spectra was performed by a standard procedure reported previously.^[^ ^45^, ^46^ ^]^ In the course of EXAFS fitting analysis, all the parameters of *R*, *σ* ^2^, and energy shift (*ΔE*) were set as variables. The electronic couplings of the present materials were investigated with PL spectroscopy (Perkin–Elmer Fluorescence Spectrometer FL8500). All the present PL spectra were collected with the excitation wavelength of 365 nm. The XPS data were measured with Thermo VG, UK, Al K_*α*_. In situ SERS spectra were taken using a Horiba Jobin Yvon LabRam Aramis with an Ar‐ion laser beam at an exciting radiation wavelength of 514.5 nm. All the electrocatalyst materials were loaded on the gold nanoparticle‐based SERS substrate.

### DFT Calculation

The authors performed spin‐polarized DFT calculations using the Vienna ab initio simulation package (VASP) code with the projector augmented wave (PAW) method.^[^ ^47^, ^48^, ^49^ ^]^ The kinetic cutoff energy for the plane wave basis set was 500 eV. For the exchange–correlation (xc) functional, the generalized gradient approximation due to Perdew, Burke, and Ernzerhof (GGA‐PBE)^[^ ^50^ ^]^ was used with the Tkatchenko and Scheffler dispersion correction via iterative Hirshfeld partitioning (TS/HI).^[^ ^51^, ^52^ ^]^ The Hubbard U correction was applied to correct the strong on‐site Coulomb interaction of d electrons of Fe and Ni atoms to all DFT calculations. U values were chosen from earlier study^[^ ^53^ ^]^ and are taken as 4.0 and 3.2 eV for the Fe and Ni atom, respectively. The Brillouin zone integration was performed using a Monkhorst‐Pack k‐grid mesh with a k‐spacing value of 0.2 Å^−1^ value. The Bader charges were converged to an integration grid spacing of within 0.005 e per atom. The atomic model of Ni−Fe‐LDH and detailed parameters are shown in Figure S20, Supporting Information. To construct the TiN−LDH nanohybrid interface model, the authors aligned the common surface lattice vectors of Ni−Fe‐LDH and TiN(001) while varying the periodicity of their surface supercells to search for an optimal interface lattice vector combination which produces the smallest possible lateral strain (≈less than 5%).^[^ ^26^ ^]^ To achieve a small (compressive) lateral strain of −4.08%, a p(1 × 1) cell of LDH on p(7 × 2) TiN(001) interface model (with 192 atoms) was chosen, see the Experimental Section of DFT calculations.

### Electrocatalytic Activity Test

The catalyst ink was obtained by dispersing 2 mg of the catalyst and 20 µL of a 5 wt% Nafion solution in a 1 mL of mixed solution of Milli‐Q water/isopropanol (4/1, v/v) by sonication for 1 h. A 10 µL drop of the prepared catalyst ink was placed onto the glassy carbon (GC) electrode (3 mm diameter, ALS Co.) and dried at 50 °C in ambient atmosphere. The obtained electrode was employed as the working electrode. A platinum wire, a saturated calomel electrode (SCE), and 1 m KOH solution were used as a counter electrode, a reference electrode, and an electrolyte, respectively. The measured potentials were normalized considering RHE as a reference. All the CV and LSV experiments were carried out with a RRDE‐3A (ALS Co.) as rotator and an IVIUM analyzer with a conventional three‐electrode cell. The electrolyte was deaerated before the measurements by purging oxygen gas for 30 min. The LSV curves were measured in a scan rate of 5 mV s^−1^ and a rotating speed of 1600 rpm. The measured potentials were normalized with respect to RHE according to the following equation: *E*_RHE_ = *E*_SCE_ + 1.0443 V (for 1 m KOH solution). The RHE calibration was done according to the previous reported method (Figure S21, Supporting Information).^[^ ^54^ ^]^ The overpotentials were calculated as *E(*overpotential) = *E*(10 mA cm^−2^)− 1.23 V. The Tafel slopes were obtained by plotting the overpotential (*η*) against log (j) curves. The EIS data were collected with the IVIUM analyzer in the frequency region of 100 kHz−100 mHz.

### Li−O_2_ Battery Test

The cathode electrodes were prepared from the materials (holey TiN NTs, Ni−Fe‐LDH, TiN−LDH‐2, and non‐holey‐TiN−LDH‐2) as follows; in a ratio of 40:45:15; each material was mixed with ketjen black and binder, poly(vinylidene fluoride‐co‐hexafluoropropylene) (HFP–PVDF) dissolved in *N*‐methyl‐2‐pyrrolidone solution to make a slurry which was then cast onto a carbon paper and heat treated at 120 °C for 5 h. The resultant electrode was cut to a 12*ϕ* size, coupled with a Li‐metal anode and microglass fiber separator wetted with a 1 m LiCF_3_SO_3_ (lithium triflouromethanesulfonate) in TEGDME (as electrolyte) assembled in a Swagelok type Li−O_2_ battery set‐up, in an inert (Ar‐filled) atmosphere. Prior to the testing, all the cells were purged with oxygen at a partial pressure of ≈0.1 mPa. CV scans were run at 5 mV s^−1^ between the voltage range 2−4.5 V and LSV scans from open circuit voltage to 5 V. Full‐range charge−discharge curves were obtained within 2−4.5 V cut‐off voltages and 100 mA g^−1^ current density. Cycle performance experiments were conducted at limited capacity of 500 mAh g^−1^ and current density of 100 mA g^−1^.

### Statistical Analysis

All the electrochemical measurements were repeated at least five times reproducing the same performance.

## Conflict of Interest

The authors declare no conflict of interest.

## Supporting information

Supporting Information

## Data Availability

Research data are not shared.

## References

[1] a) X. Jin , D. A. Agyeman , S. Kim , Y. H. Kim , M. G. Kim , Y.‐M. Kang , S.‐J. Hwang , Nano Energy 2020, 67, 104192;

[2] H. Yu , T. Zhou , Z. Wang , Y. Xu , X. Li , L. Wang , Angew. Chem. Int. Ed. 2021, 60, 12027. 10.1002/anie.202101019 33559316

[3] M. Gerosa , F. Gygi , M. Govoni , G. Galli , Nat. Mater. 2018, 17, 1122. 30374203 10.1038/s41563-018-0192-4

[4] Y. Gao , J. Lu , J. Xia , G. Yu , ACS Appl. Mater. Interfaces 2020, 12, 12706. 32077683 10.1021/acsami.9b21122

[5] Y. Zhang , L. Tao , C. Xie , D. Wang , Y. Zou , R. Chen , Y. Wang , C. Jia , S. Wang , Adv. Mater. 2020, 32, 1905923. 10.1002/adma.201905923 31930593

[6] a) N. Kim , T.‐H. Gu , D. Shin , X. Jin , H. Shin , M. G. Kim , H. Kim , S.‐J. Hwang , ACS Nano 2021, 15, 8306; 33861569 10.1021/acsnano.0c09217

[7] a) Z. Xiao , C. Xie , Y. Wang , R. Chen , S. Wang , J. Energy Chem. 2021, 53, 208;

[8] a) X. Sun , X. Zhang , Y. Xie , Matter 2020, 2, 842;

[9] S. Kahaharai , T. lijima , S. Ogawa , S. Suzuki , S.‐L. Li , K. Tsukagoshi , S. Sato , N. Yokoyama , ACS Nano 2013, 7, 5694. 23786356 10.1021/nn401992q

[10] D. Chen , M. Qiao , Y.‐R. Lu , L. Hao , D. Liu , C.‐L. Dong , Y. Li , S. Wang , Angew. Chem. Int. Ed. 2018, 57, 8691. 10.1002/anie.201805520 29771458

[11] L. Bao , L. Chang , L. Yao , W. Meng , Q. Yu , X. Zhang , X. Liu , X. Wang , W. Chen , X. Li , New J. Chem. 2021, 45, 3546.

[12] a) X. Jin , T.‐H. Gu , K.‐G. Lee , M. J. Kim , M. S. Islam , S.‐J. Hwang , Coord. Chem. Rev. 2020, 415, 213280;

[13] a) S. M. Oh , S. B. Patil , X. Jin , S.‐J. Hwang , Chem. Eur. J. 2018, 24, 4757; 29071739 10.1002/chem.201704284

[14] Y. Zhang , L. Guo , L. Tao , Y. Lu , S. Wang , Small Methods 2019, 3, 1800406.

[15] X. Jin , S. Y. Son , M. G. Kim , S.‐J. Hwang , Nano Energy 2020, 78, 105255.

[16] H. Shin , H.‐i. Kim , D. Y. Chung , J. M. Yoo , S. Weon , W. Choi , Y.‐E. Sung , ACS Catal. 2016, 6, 3914.

[17] R.‐Q. Zhang , T.‐H. Lee , B.‐D. Yu , C. Stampfl , A. Soon , Phys. Chem. Chem. Phys. 2012, 14, 16552. 22772941 10.1039/c2cp41392b

[18] T. Lee , B. Delley , C. Stampfl , A. Soon , Nanoscale 2012, 4, 5183. 22806505 10.1039/c2nr31266b

[19] J. Shen , P. Zhang , R. Xie , L. Chen , M. Li , J. Li , B. Ji , Z. Hu , J. Li , L. Song , Y. Wu , X. Zhao , ACS Appl. Mater. Interfaces 2019, 11, 13545. 30892865 10.1021/acsami.8b22260

[20] Q. Wang , L. Chen , S. Guan , X. Zhang , B. Wang , X. Cao , Z. Yu , Y. He , D. G. Evans , J. Feng , D. Li , ACS Catal. 2018, 8, 3104.

[21] Y. Zhao , L. Zheng , R. Shi , S. Zhang , X. Bian , F. Wu , X. Cao , G. I. N. Waterhouse , T. Zhang , Adv. Energy Mater. 2020, 10, 2002199.

[22] S. L. Moffitt , Q. Ma , D. B. Buchholz , R. P. H. Chang , M. J. Bedzyk , T. O. Mason , J. Phys.: Conf. Ser. 2016, 712, 012116.

[23] W. Tang , E. Sanville , G. Henkelman , J. Phys.: Condens. Matter 2009, 21, 084204. 21817356 10.1088/0953-8984/21/8/084204

[24] Y. H. Kim , X. Jin , S.‐J. Hwang , J. Mater. Chem. A 2019, 7, 10971.

[25] W. Tang , E. Sanville , G. Henkelman , J. Phys.: Condens. Matter 2009, 21, 084204. 21817356 10.1088/0953-8984/21/8/084204

[26] P. Lazić , Comput. Phys. Commun. 2015, 197, 324.

[27] S. Chen , J. Duan , M. Jaroniec , S.‐Z. Qiao , Adv. Mater. 2014, 26, 2925. 24510748 10.1002/adma.201305608

[28] Q. Cheng , Z. Chen , Int. J. Electrochem. Sci. 2013, 8, 8282.

[29] Q. Zhang , Z. D. Wei , C. Liu , X. Liu , X. Q. Qi , S. G. Chen , W. Ding , Y. Ma , F. Shi , Y. M. Zhou , Int. J. Hydrogen Energy 2012, 37, 822.

[30] M. Gong , Y. Li , H. Wang , Y. Liang , J. Z. Wu , J. Zhou , J. Wang , T. Regier , F. Wei , H. Dai , J. Am. Chem. Soc. 2013, 135, 8452. 23701670 10.1021/ja4027715

[31] Y. Wang , R. Ohnishi , E. Yoo , P. He , J. Kubota , K. Domen , H. Zhou , J. Mater. Chem. 2012, 22, 15549.

[32] S. Chitravathi , S. Kumar , N. Munichandraiah , RSC Adv. 2016, 6, 103106.

[33] H. Lee , O. Kwon , K. Choi , L. Zhang , J. Zhou , J. Park , J.‐W. Yoo , J.‐Q. Wang , J. H. Lee , G. Kim , ACS Catal. 2020, 10, 4664.

[34] D. Zhou , Z. Cai , Y. Jia , X. Xiong , Q. Xie , S. Wang , Y. Zhang , W. Liu , H. Duan , X. Sun , Nanoscale Horiz.. 2018, 3, 532. 32254139 10.1039/c8nh00121a

[35] C. Peng , N. Ran , G. Wan , W. Zhao , Z. Kuang , Z. Lu , C. Sun , J. Liu , L. Wang , H. Chen , ChemSusChem 2020, 13, 811. 31802649 10.1002/cssc.201902841

[36] Z. Lu , W. Xu , W. Zhu , Q. Yang , X. Lei , J. Liu , Y. Li , X. Sun , X. Duan , Chem. Commun. 2014, 50, 6479. 10.1039/c4cc01625d 24817324

[37] Z. Qiu , C.‐W. Tai , G. A. Niklasson , T. Edvinsson , Energy Environ. Sci. 2019, 12, 572.

[38] W. Luo , C. Jiang , Y. Li , S. A. Shevlin , X. Han , K. Qiu , Y. Cheng , Z. Guo , W. Huang , J. Tang , J. Mater. Chem. 2017, 5, 2021.

[39] X. Jin , M. Park , S.‐J. Shin , Y. Jo , M. G. Kim , H. Kim , Y.‐M. Kang , S.‐J. Hwang , Small 2020, 16, 1903265. 10.1002/smll.201903265 31490620

[40] S. Hirai , K. Morita , K. Yasuoka , T. Shibuya , Y. Tojo , Y. Kamihara , A. Miura , H. Suzuki , T. Ohno , T. Matsuda , S. Yagi , J. Mater. Chem. A 2018, 6, 15102.

[41] J. Li , C. Shu , C. Liu , X. Chen , A. Hu , J. Long , Small 2020, 16, 2001812. 10.1002/smll.202001812 32431080

[42] Y. Pan , H. Ren , H. Du , F. Cao , Y. Jiang , H. Du , D. Chu , J. Mater. Chem. A 2018, 6, 22497.

[43] Z. Chang , J. Xu , X. Zhang , Adv. Energy Mater. 2017, 7, 1700875.

[44] K. Song , D. A. Agyeman , M. Park , J. Yang , Y.‐M. Kang , Adv. Mater. 2017, 29, 1606572. 10.1002/adma.201606572 28940885

[45] a) S.‐J. Hwang , H.‐S. Park , J.‐H. Choy , G. Campet , Chem. Mater. 2000, 12, 1818;

[46] D. H. Park , S.‐H. Lee , T. W. Kim , S. T. Lim , S.‐J. Hwang , Y. S. Yoon , Y.‐H. Lee , J.‐H. Choy , Adv. Funct. Mater. 2007, 17, 2949.

[47] G. Kresse , Phys. Rev. B 1993, 47, 558. 10.1103/physrevb.47.558 10004490

[48] G. Kresse , J. Furthmülle , Phys. Rev. B 1996, 54, 11169. 10.1103/physrevb.54.11169 9984901

[49] G. Kresse , D. Joubert , Phys. Rev. B 1999, 59, 1758.

[50] J. P. Perdew , K. Burke , M. Ernzerhof , Phys. Rev. Lett. 1997, 78, 1396. 10.1103/PhysRevLett.77.3865 10062328

[51] A. Tkatchenko , M. Scheffler , Phys. Rev. Lett. 2009, 102, 073005. 19257665 10.1103/PhysRevLett.102.073005

[52] T. Bucko , S. Lebègue , J. Hafner , J. G. Ángyán , J. Chem. Theory Comput. 2013, 9, 4293. 26589148 10.1021/ct400694h

[53] A. C. Thenuwara , N. H. Attanayake , J. Yu , J. P. Perdew , E. J. Elzinga , Q. Yan , D. R. Strongin , J. Phys. Chem. B 2018, 122, 847. 28880559 10.1021/acs.jpcb.7b06935

[54] G. Fu , Y. Wang , Y. Tang , K. Zhou , J. B. Goodenough , J.‐M. Lee , ACS Mater. Lett. 2019, 1, 123.

