# Supplementary material for: In Situ Defect Engineering Route to Optimize the Cationic Redox Activity of Layered Double Hydroxide Nanosheet via Strong Electronic Coupling with Holey Substrate
Source: Adv Sci (Weinh). 2021 Oct 28;9(1):2103368. doi: 10.1002/advs.202103368 (PMC8728845; doi:10.1002/advs.202103368)
Supplement: Supplementary file 1 — Supporting Information [file ADVS-9-2103368-s001.pdf]

ADVANCED SCIENCE
Open Access

Supporting Information

for *Adv. Sci.*, DOI: 10.1002/advs.202103368

In Situ Defect Engineering Route to Optimize the Cationic Redox Activity of Layered Double Hydroxide Nanosheet via Strong Electronic Coupling with Holey Substrate

Xiaoyan Jin, Taehun Lee, Wilson Tamakloe, Sharad B. Patil, Aloysius Soon, Yong-Mook Kang,* and Seong-Ju Hwang**

© Copyright 2020. WILEY-VCH GmbH.

Supporting Information

In Situ Defect Engineering Route to Optimize the Cationic Redox Activity of Layered Double Hydroxide Nanosheet via Strong Electronic Coupling with Holey Substrate

Xiaoyan Jin, Taehun Lee, Wilson Tamakloe, Sharad B. Patil, Aloysius Soon, Yong-Mook Kang,* and Seong-Ju Hwang**

Dr. X. Jin, Prof. S.-J. Hwang
Department of Materials Science and Engineering, College of Engineering,
Yonsei University, Seoul 03722, Republic of Korea
E-mail: hwangsju@yonsei.ac.kr

Dr. T. Lee, Prof. A. Soon
Center for Artificial Synesthesia Materials Discovery, Department of Materials Science and Engineering, Yonsei University, Seoul 03722, Republic of Korea
E-mail: aloysius.soon@yonsei.ac.kr

W. Tamakloe, Prof. Y.-M. Kang
Department of Materials Science and Engineering,
Korea University, Seoul 02841, Republic of Korea
E-mail: dake1234@korea.ac.kr

Dr. S. B. Patil
Department of Chemistry and Nanoscience, College of Natural Sciences,
Ewha Womans University, Seoul 03760, Republic of Korea

Prof. Y.-M. Kang
KU-KIST Graduate School of Converging Science and Technology,
Korea University, Seoul 02841, Republic of Korea

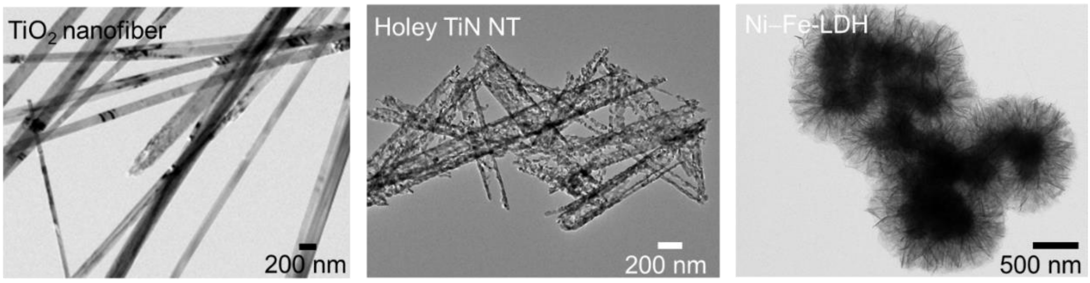

Figure S1. Transmission electron microscopy (TEM) images of TiO_2 nanofiber, holey TiN nanotubes (NTs), and Ni–Fe-layered double hydroxide (LDH) NSs.

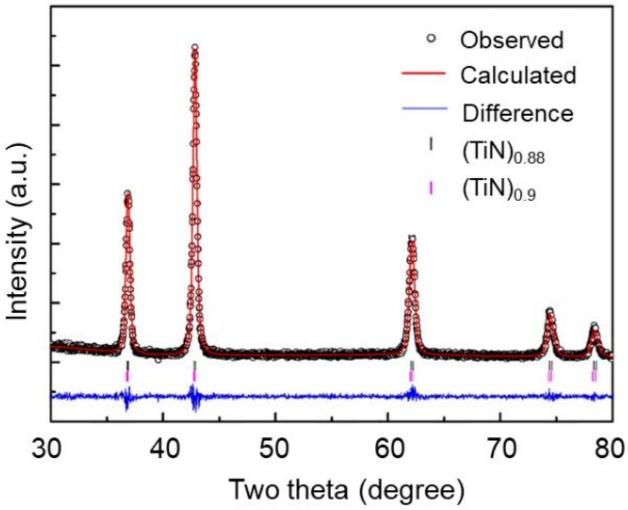

Figure S2. Powder X-ray diffraction (XRD) pattern of the holey TiN NTs and Rietveld refinement result.

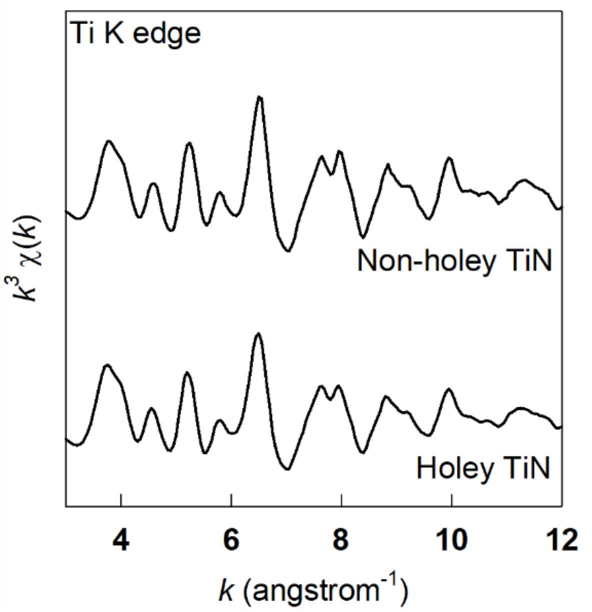

Figure S3. k^3-weighted Ti K-edge extended X-ray absorption fine structure (EXAFS) data of holey TiN NTs and non-holey TiN reference.

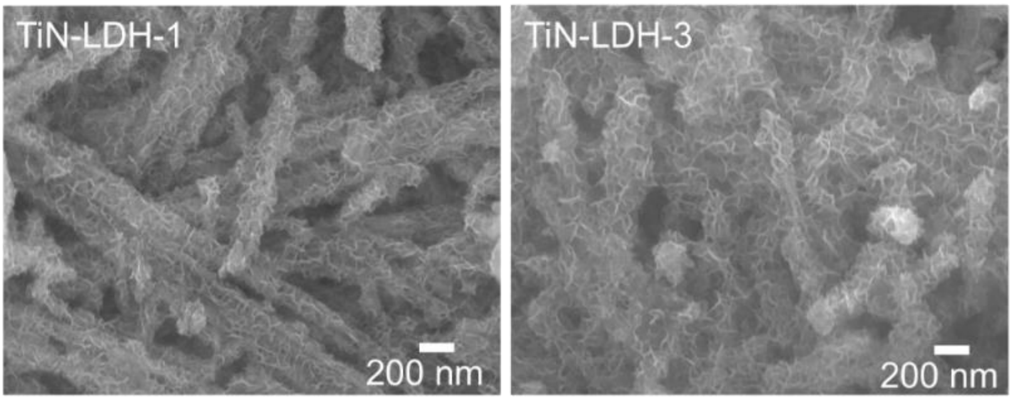

Figure S4. Field emission-scanning electron microscopy (FE-SEM) images of TiN−LDH-1 and TiN−LDH-3.

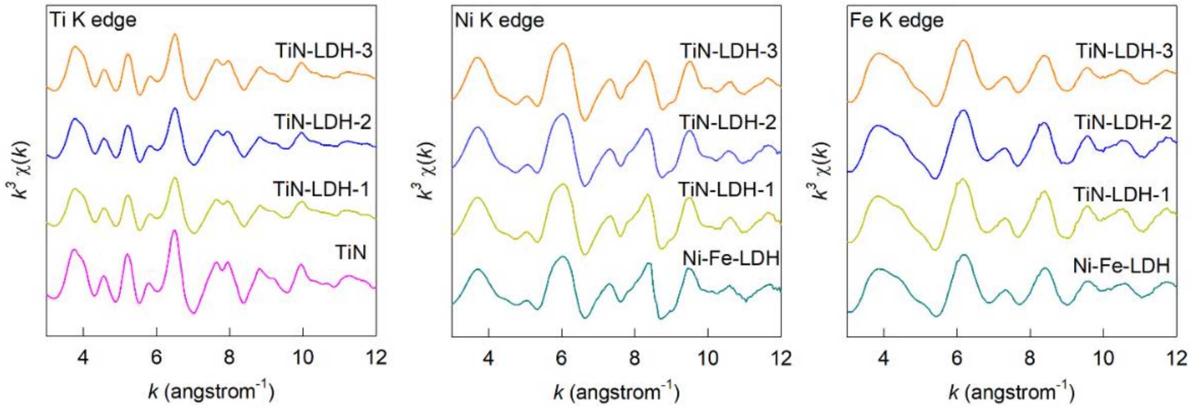

Figure S5. k^3-weighted Ti K-edge, Ni K-edge, and Fe K-edge EXAFS oscillations of TiN–LDH nanohybrids and Ni–Fe-LDH.

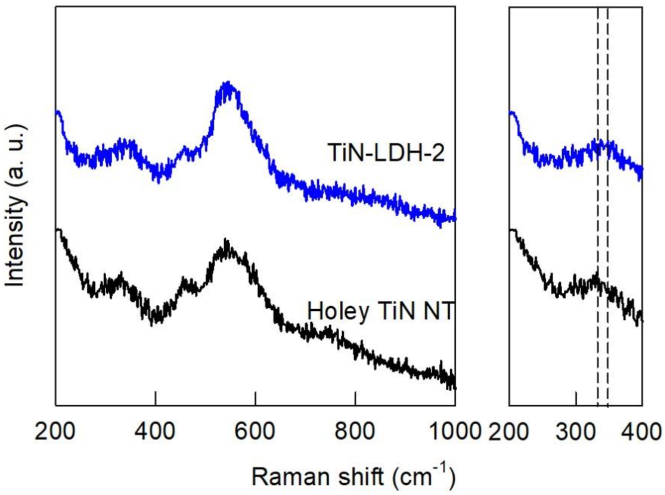

Figure S6. Micro-Raman spectra of holey TiN NT and TiN–LDH-2.

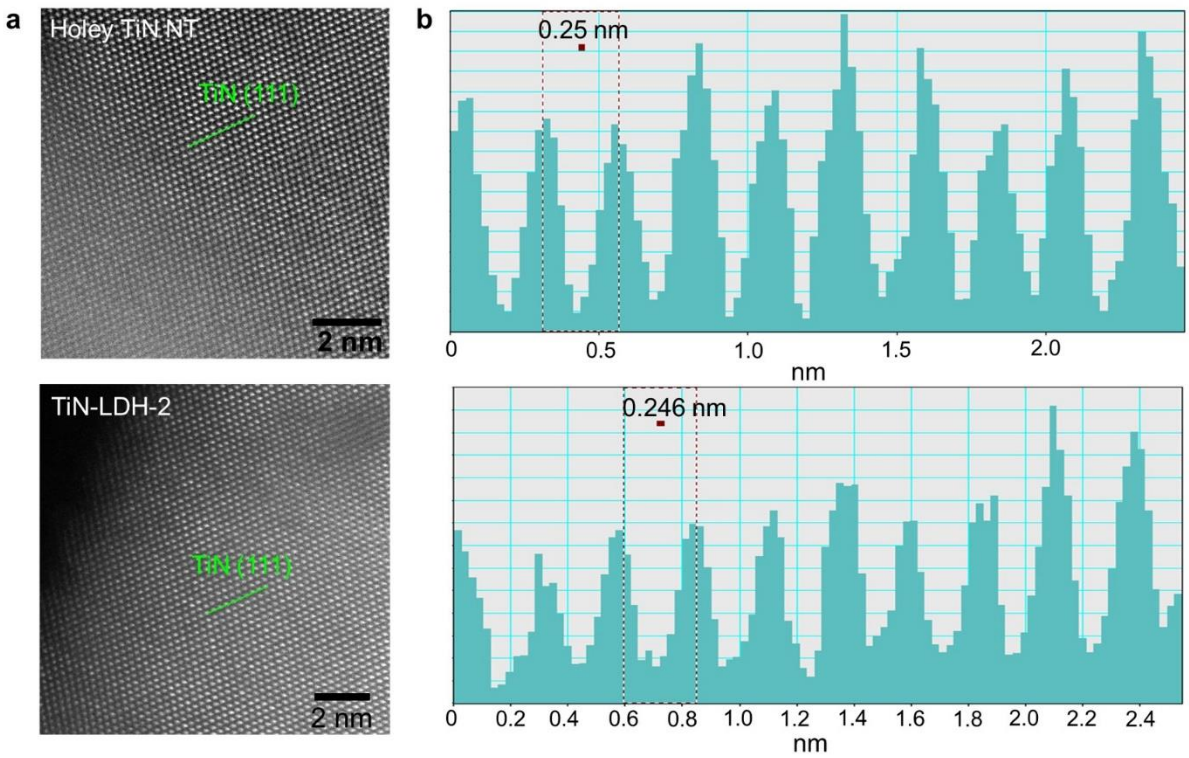

Figure S7. (a) Cs-corrected scanning transmission electron microscopy (STEM) images and (b) lattice distance profiles of holey TiN NT and TiN–LDH-2.

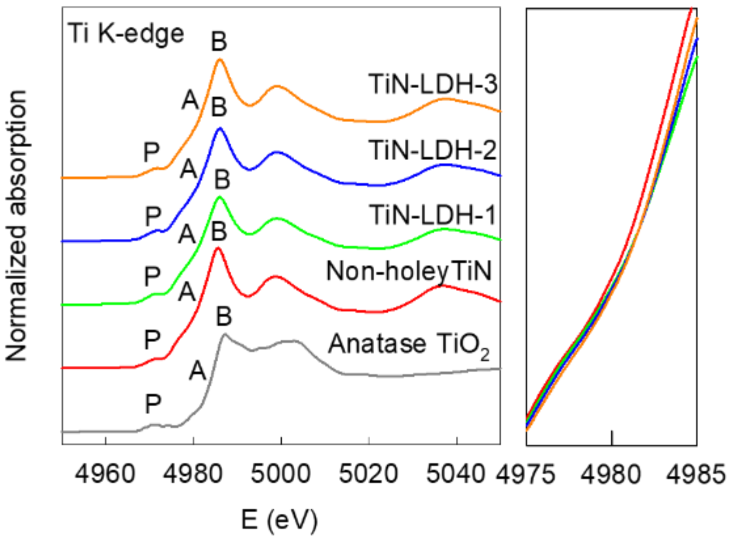

Figure S8. Ti K-edge X-ray absorption near-edge structure (XANES) spectra of TiN–LDH nanohybrids, non-holey TiN, and anatase TiO_2.

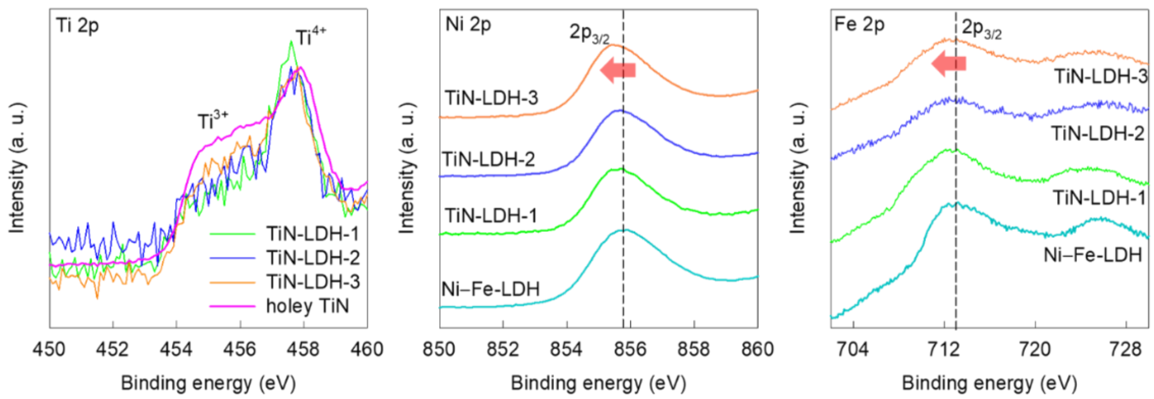

Figure S9. X-ray photoelectron spectra (XPS) of TiN–LDH nanohybrids, holey TiN, and Ni–Fe-LDH.

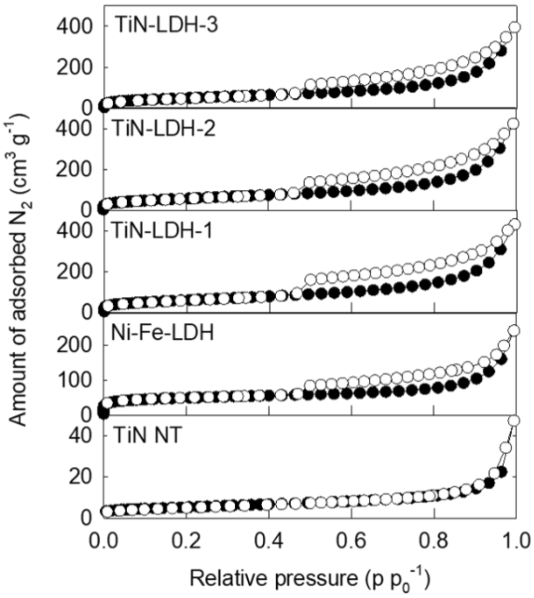

Figure S10. N_2 adsorption–desorption isotherms of TiN–LDH nanohybrids, Ni–Fe-LDH, and TiN NT.

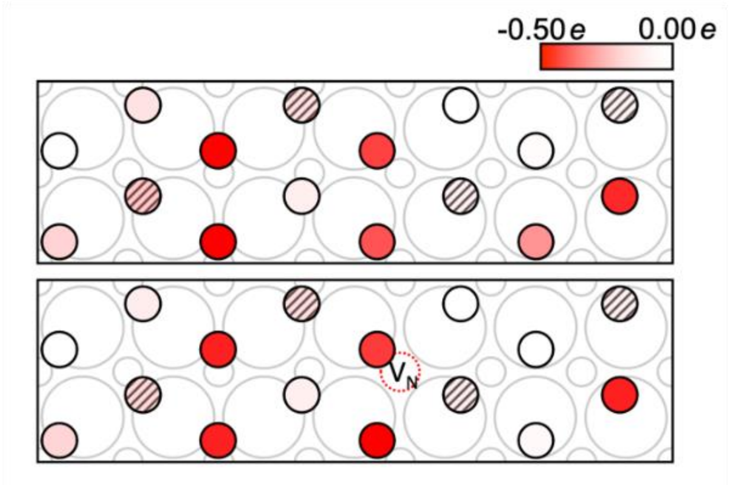

Figure S11. Degree of charge accumulation in Ni (circles) and Fe (hashed circles) atoms in the LDH layer with (lower panel) and without (upper panel) nitrogen vacancies.

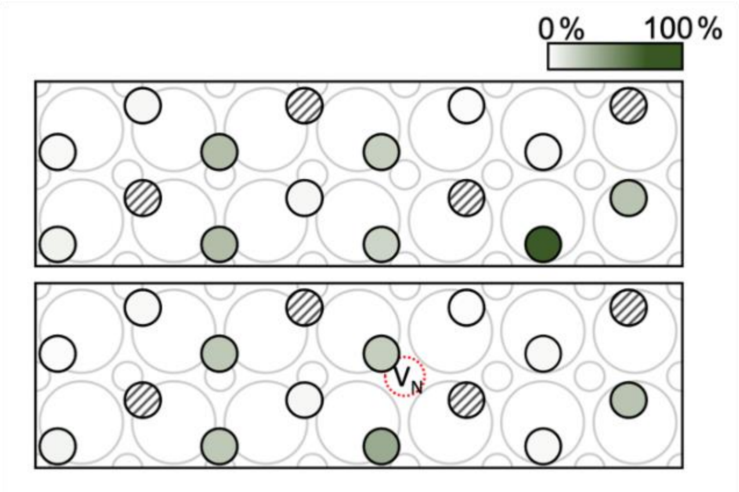

Figure S12. Degree of charge accumulation in Ni (circles) and Fe (hashed circles) atoms in the LDH layer with (lower panel) and without (upper panel) nitrogen vacancies.

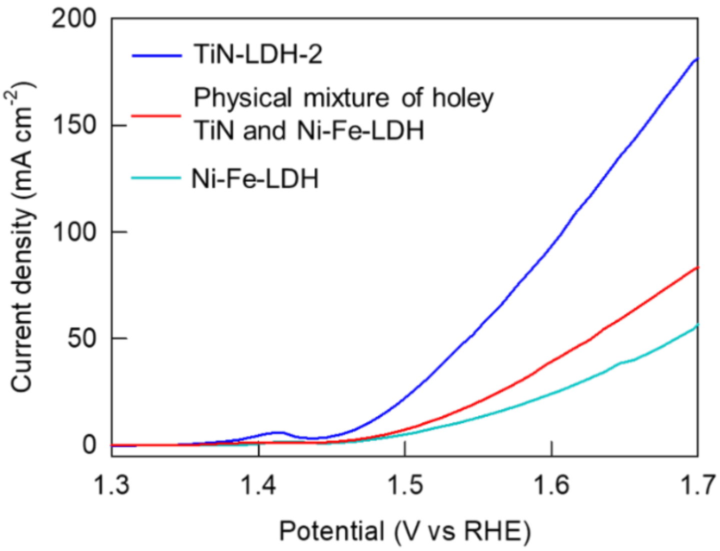

Figure S13. Linear sweep voltammetry (LSV) curves of oxygen evolution reaction (OER) for physical mixture of Ni–Fe-LDH and TiN NTs.

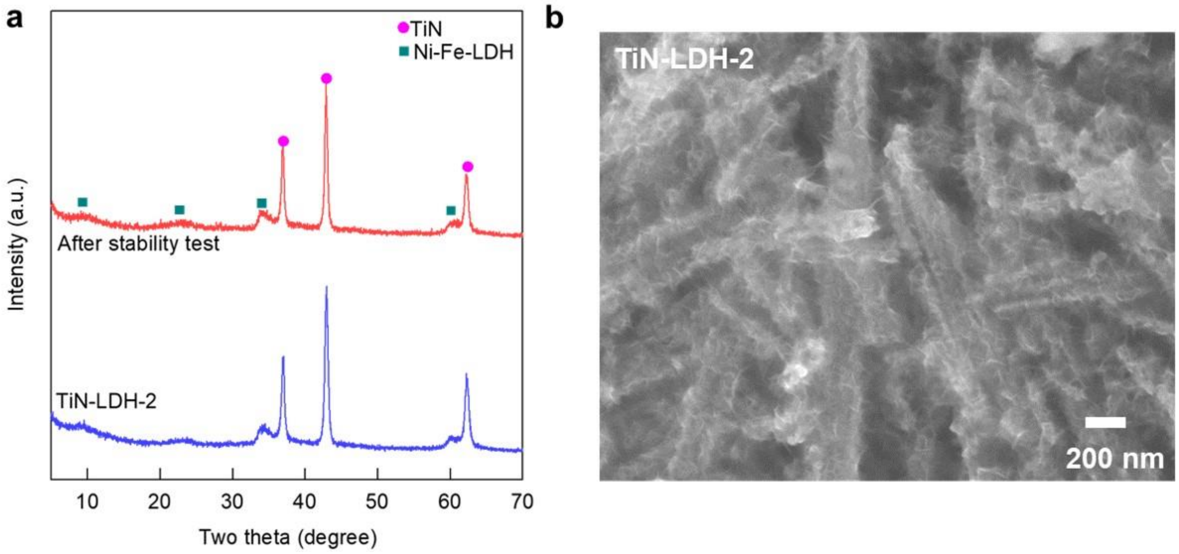

Figure S14. (a) Powder XRD pattern and (b) FE-SEM image of TiN–LDH-2 after the stability test.

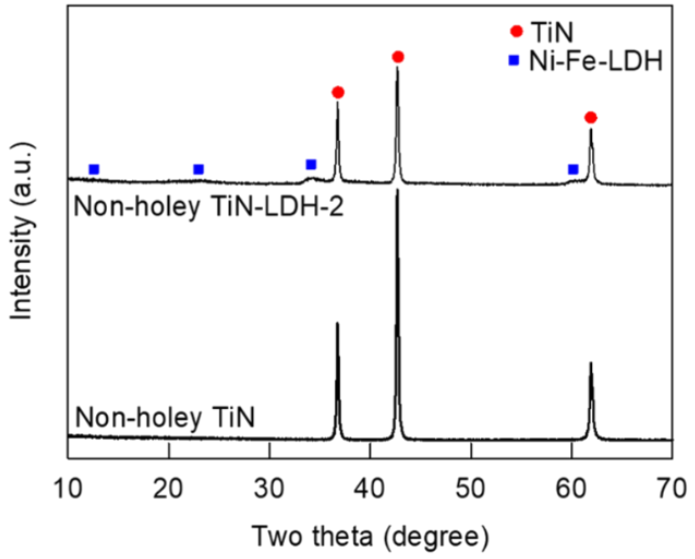

Figure S15. Powder XRD patterns of non-holey TiN–LDH-2.

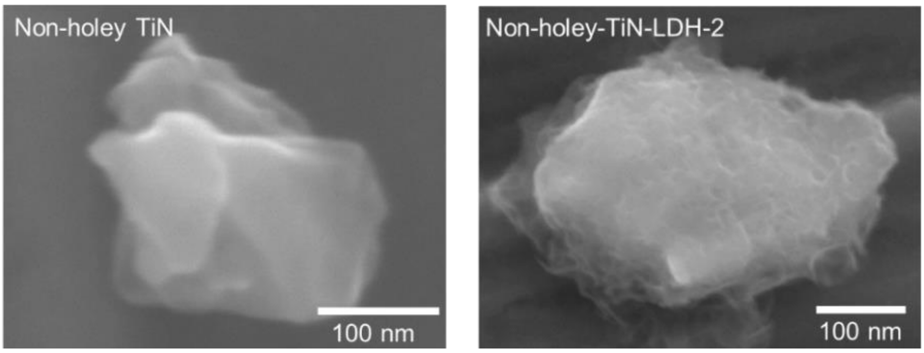

Figure S16. FE-SEM images of non-holey TiN–LDH-2.

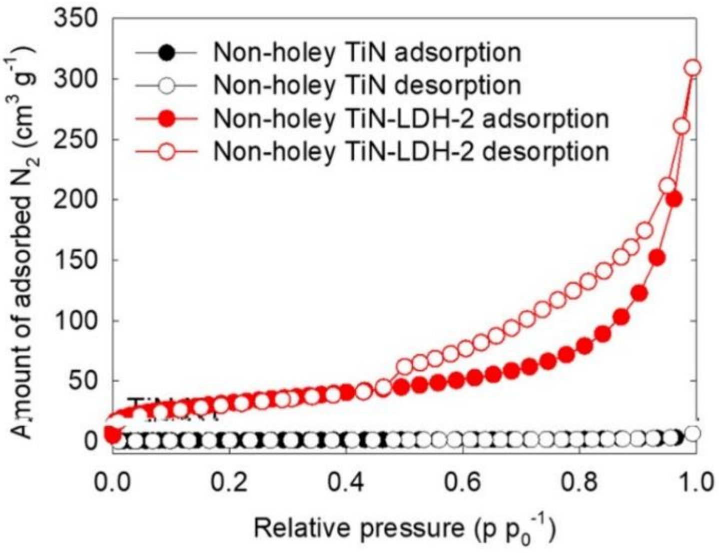

Figure S17. N_2 adsorption–desorption isotherms of non-holey TiN–LDH-2.

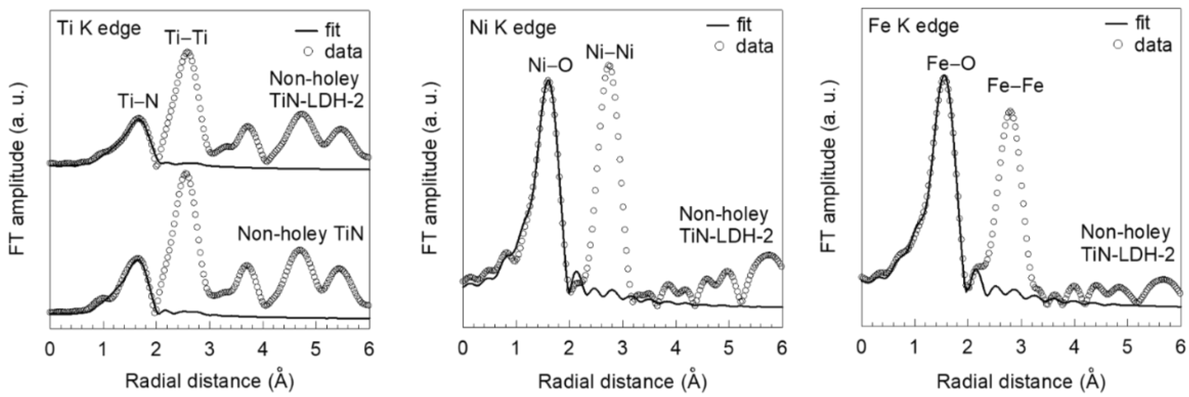

Figure S18. Fourier transformed (FT)-EXAFS spectra of non-holey TiN–LDH-2.

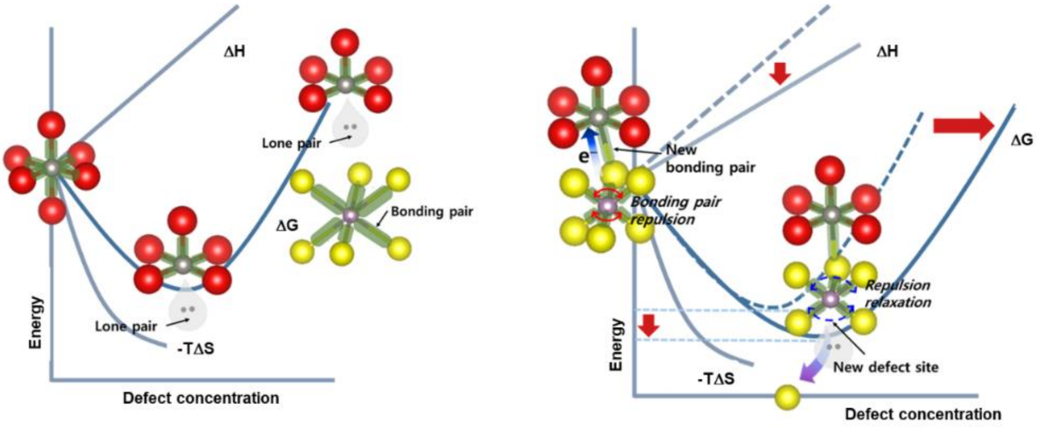

Figure S19. Schematic model for the effect of intimate hybridization on the entropy and enthalpy changes in defect formation.

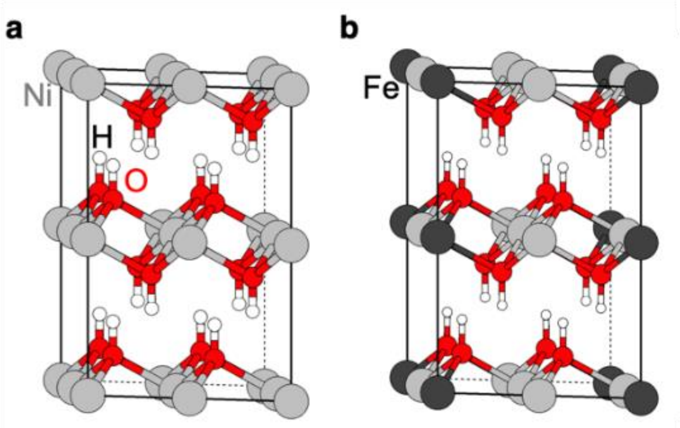

Figure S20. Atomic structures of (a) $Ni(OH)_2$ and (b) Ni–Fe-LDH. The calculated lattice constants, a_0 and c_0 for brucite $Ni(OH)_2$ (with a space group *P*3m1) are 3.13 and 4.45 Å, respectively. This is in better agreement with previous experiments (3.13 and 4.63 Å) than previously reported theoretical values (3.22 and 4.71 Å).[1] The calculated magnetic moment (M) for the Ni atom in $Ni(OH)_2$ is 1.712 μB, with an anti-ferromagnetic magnetic (AFM) ordering. To search for the ground state structures of $Ni_{0.75}Fe_{0.25}(OH)_2$ (i.e., Ni–Fe-LDH) solid solution, different configurational structures are generated within a p(2×2×2) supercell of $Ni(OH)_2$ using the site occupancy disorder (SOD) program.[2] The calculated structural parameters a0 and c0 for $Ni_{0.75}Fe_{0.25}(OH)_2$ are 6.32 and 8.95 Å, respectively. The magnetic moments for the Ni and Fe atoms are found to be 1.716 and 3.762 μB, respectively, assuming an AFM ordering.

To minimize the lattice mismatch at the interface, a strained $\begin{pmatrix} 4 & 8 \\ 0 & 1 \end{pmatrix}$ supercell of $Ni_{0.75}Fe_{0.25}(OH)_2$ (as constructed from a p(1 × 1) cell of $Ni(OH)_2$) is supported on a p(7 × 2) supercell of TiN(001), resulting in a relatively small compressive strain (less than 5%) in the LDH layer. The interface model is constructed with one monolayer of LDH and four atomic layers of TiN. For geometry optimization of this interface, the bottom two atomic layers of TiN(001) are kept fixed to that in bulk TiN. A vacuum region of 15 Å (with the standard dipole correction) along the perpendicular direction of the interface is adopted to avoid unphysical interactions between repeated images.

To assess the thermodynamic stability of the nitrogen vacancy in different structures, we define the formation energy of the nitrogen defect, E^{form} as

$$E^{\text{form}} = \frac{1}{N_D}\left(E_{\text{Slab}}^{\text{TiN-LDH}} - E_{\text{Clean}}^{\text{TiN-LDH}} - \Delta N_{\text{D}}\mu_N\right) \qquad (1),$$

where $E_{\text{Slab}}^{\text{TiN-LDH}}$ and $E_{\text{Clean}}^{\text{TiN-LDH}}$ are the DFT calculated ground state total energies for the defect-containing and defect-free TiN, respectively. Here, N_{D} is the number of nitrogen vacancies at the outermost layer of TiN and μ_{N} is taken as the atomic chemical potential of nitrogen. In this work, the chemical reference state for the nitrogen atom is taken as the energy to decompose bulk TiN to its elemental components Ti and N, as defined under the nitrogen-lean condition. The PBE+TS calculated E^{form} for TiN(001) is −0.23 eV which agrees with previous reported PBE value of –0.37 eV.[3,4]

Figure S21. LSV curve measured at a scan rate of 1 mV s^{-1}. The average potential under the zero current was taken for the thermodynamic potential of the hydrogen electrode reactions.

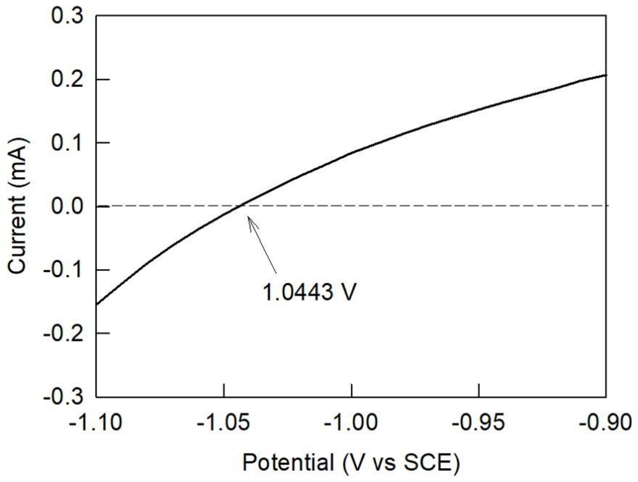

Table S1. Refined structural parameters and fitting factors of holey TiN NTs.

Parameters		Value
Lattice constant:	$TiN_{0.88}$	4.22452(58)
a (Å)	$TiN_{0.9}$	4.22868(35)
Weight fraction (%)	$TiN_{0.88}$	71.95
	$TiN_{0.9}$	28.05
R_{wp} (%)		11.97
R_{exp} (%)		8.74
R_p (%)		8.57
χ^2		1.87
R_F (%)		1.96

Table S2. Calculated effective Bader charges (Δq_{eff}) of TiN, LDH layers, and LDH/TiN interfaces, normalized by the number of Ti, N, Fe, Ni, O, and H atoms in the corresponding systems. The Bader charges are analyzed by averaging the values of a) outermost Ti and N atoms of the TiN layer, b) the nearest Ti atoms to the nitrogen vacancy, and c) the other remaining Ti atoms, respectively. V_N: nitrogen vacancies.

Structure	Ti[a)] (*e*)	Ti[b)] (*e*)	Ti[c)] (*e*)	N[a)] (*e*)	Fe (*e*)	Ni (*e*)	O (*e*)	H (*e*)
TiN(001)	+1.74			–1.68				
TiN(001)+V_N	+1.66	+1.49	+1.73	–1.69				
Ni–Fe-LDH					+1.41	+1.20	–1.19	+0.56
Ni–Fe-LDH/TiN(001)	+1.83			–1.62	+1.36	+0.99	–1.19	+0.56
Ni–Fe-LDH/TiN(001)+V_N	+1.76	+1.67	+1.80	–1.63	+1.36	+1.01	–1.20	+0.56

REFERENCES

[1] P. Hermet, L. Gourrier, J.-L. Bantignies, D. Ravot, T. Michel, S. Deabate, P. Boulet, F. Henn, *Phys. Rev. B* **2011**, *84*, 235211.

[2] R. Grau-Crespo, S. Hamad, S. C. R. A. Catlow, N. H. Leeuw, *Phys.: Condens. Matter* **2007**, *19*, 256201.

[3] T. Lee, B. Delley, C. Stampfl, A. Soon, *Nanoscale* **2012**, *4*, 5183.

[4] R.-Q. Zhang, T.-H. Lee, B.-D. Yu, C. Stampfl, A. Soon, *Phys. Chem. Chem. Phys.* **2012**, *14*, 16552.
